# Aberrant DNA methylation of the *toll-like receptors* 2 and 6 genes in patients with obstructive sleep apnea

**DOI:** 10.1371/journal.pone.0228958

**Published:** 2020-02-18

**Authors:** Kuo-Tung Huang, Yung-Che Chen, Chia-Cheng Tseng, Huang-Chih Chang, Mao-Chang Su, Ting-Ya Wang, Yong-Yong Lin, Yi-Xin Zheng, Jen-Chieh Chang, Chien-Hung Chin, Chang-Chun Hsiao, Meng-Chih Lin

**Affiliations:** 1 Division of Pulmonary and Critical Care Medicine, Department of Medicine, Kaohsiung Chang Gung Memorial Hospital and Chang Gung University College of Medicine, Kaohsiung, Taiwan; 2 Sleep Center, Kaohsiung Chang Gung Memorial Hospital and Chang Gung University College of Medicine, Kaohsiung, Taiwan; 3 Graduate Institute of Clinical Medical Sciences, College of Medicine, Chang Gung University, Division of Pulmonary and Critical Care Medicine, Kaohsiung Chang Gung Memorial Hospital, Kaohsiung, Taiwan; 4 Department of Nursing, Meiho University, Pingtung, Taiwan; 5 Chang Gung University of Science and Technology, Chia-yi, Taiwan; Brigham and Women's Hospital and Harvard Medical School, UNITED STATES

## Abstract

Obstructive sleep apnea (OSA) is a syndrome leading to chronic intermittent hypoxia, and the up-regulation of toll-like receptors (TLR) 2 and 6 on peripheral blood cells has been reported. We hypothesized that DNA methylation in *TLR2* and *TLR6* genes may play a role in the development of OSA and its excessive daytime sleepiness (EDS) phenotype. DNA methylation over 28 cytosine-phosphate-guanine (CpG) sites of the *TLR2* promoter region and 3 CpG sites of the *TLR6* gene body, and their protein expressions were measured by using pyrosequencing and ELISA methods in 18 heathy subjects (HS) and 58 patients with severe OSA (divided into 18 non-EDS and 40 EDS group). Patients with severe OSA had higher DNA methylation levels over five CpG sites (#1, #2, #3, #25 and #28) and lower DNA methylation levels over CpG site #18 of the *TLR2* promoter region, higher DNA methylation levels over two CpG sites (#1 and #3) of the *TLR6* gene body, and higher protein expressions of TLR6 than HS. The CpG site #2 of the *TLR6* gene body was hypermethylated in severe OSA patients with EDS. Both DNA methylation levels over CpG site #1 of the *TLR6* gene body and protein expressions of TLR6 were reduced after more than 6 months of nasal CPAP treatment in seven selected patients. Aberrant DNA methylation of the *TLR2* promoter region and *TLR6* gene body are associated with the consequence of severe OSA and its EDS phenotype.

## Introduction

Obstructive sleep apnea (OSA) is a syndrome characterized by repetitive upper airway collapse during sleep, leading to chronic intermittent hypoxia, sleep fragmentation, oxidative stress and the damage similar to that caused by ischemia-reperfusion injury [1, 2]. Indeed, intermittent hypoxia (IH) activates a number of signaling pathways that are involved in oxygen sensing, oxidative stress, metabolism, catecholamine biosynthesis, and immune responsiveness. The cumulative effect of these processes over time can undermine cell integrity and lead to a decline in its function, cell injury and cell death [3]. These consequences not only result in excessive daytime sleepiness (EDS) and impaired cognitive function, but are also correlated with cardiovascular morbidities such as stroke, ischemic heart disease (IHD), congestive heart failure (CHF), and arrhythmias [4–7].

DNA methylation is a heritable, tissue-specific, and reversible gene regulatory process that is highly modified in response to environmental factors, including intermittent hypoxia [8, 9]. DNA methylation occurs at position 5 of the pyrimidine ring of cytosines in the context of the cytosine followed by guanine dinucleotide sequence (CpG) forming the basis of epigenetic mechanisms modulating gene expressions by inhibition of the binding of transcription factors (TF) at the promoter regions. The negative correlation between gene expression and the DNA methylation at the promoter regions is well-established, whereas recent studies have proven that DNA methylation status in the gene body shows a positive correlation with gene expression through alternative splicing [10–14]. DNA methylation does not only affect TF binding leading to de-regulated gene expression. More importantly, there is a crosstalk between DNA methylation and histone modifications which directly affects transcriptional gene activity.

Although aberrant DNA methylation in the promoter regions of several inflammation-related genes such as *IL1R2* (interleukin 1 receptor 2), *AR (*androgen receptor), *NPR2* (natriuretic peptide receptor 2), and *SP140* (speckled protein 140) genes have been reported in patients with OSA [15], little is known about the role of DNA methylation over the *TLR* genes in the development of OSA and its clinical phenotypes. In our previous study, we found co-upregulation of TLR 2 and 6 on peripheral blood neutrophils and mononuclear cells in patients with OSA, and these changes could be reversed after continuous positive airway pressure (CPAP) treatment [16, 17]. It has been demonstrated that hypoxia inducible factor (HIF)-1α coordinates selective induction of both *TLR2* and *TLR6* during persistent hypoxia [18], and the intermittent hypoxia led to greater than two fold upregulation of the *TLR2* in healthy volunteers has also reported[19]. The down-regulation of TLR2 expressions through aberrant DNA methylation of certain CpG sites over *TLR2* promoter region has been demonstrated in patients with active pulmonary tuberculosis (TB) disease and cystic fibrosis [20]. In this study, we hypothesized that DNA methylation in the promoter region of *TLR2* and in the *TLR6* gene body might play a role in the development of severe OSA and the EDS phenotype.

## Materials and methods

### Subjects

This study was approved by the Institutional Review Board of Chang Gung Memorial Hospital, Taiwan (certificate number: 102-3887B). The participants were recruited from both Sleep and Health Examination Centers of Kaohsiung Chang Gung Memorial Hospital from February 2014 through February 2017. Informed consent was obtained from each subject participating in the study. Adults (aged 20 to 65 years) who were diagnosed as healthy subject (HS) with primary snoring (defined as Apnea-hypopnea index(AHI) < 5 and were free of other sleep disorder) or severe OSA (defined as AHI >30) after full-night polysomnographic studies in our sleep laboratory were included. The exclusion criteria were ongoing infections, autoimmune disease, use of immunosuppressive agent in the past 6 months, narcolepsy, severe obesity (body mass index [BMI] ≥ 35 kg/m2), and those with a BMI < 21 kg/m2. Patients with severe OSA were further divided into two subgroups according to the Epworth sleepiness scale (ESS): OSA with ESS≦10 (non-EDS group) and OSA with ESS>10 (EDS group). Seven patients with severe OSA who had been under nasal CPAP management at least 4 hours per night for more than 6 months were included for further comparison.

### Polysomnography

Body height, body weight, and BMI were measured prior to the overnight polysomnographic study. Subjective sleepiness was assessed using the ESS, a 24-point questionnaire comprised of 8 questions, each with a 0–3 scale that assesses a subject’s tendency to fall asleep during various situations, where a higher score indicates increased sleepiness[21, 22]. The completed polysomnography examination, including electroencephalography, electrooculography, chin and anterior tibial electromyography, respiratory effort detectors, nasal/oral flow sensors, and pulse oximetry, was performed using a standardized commercial device (Sandman SD32+TM Digital Amplifier [Embla, Colorado, U.S.A.]).

All subjects completed their polysomnographic study with at least 4 hours of total sleep time as indicated by electroencephalography. Sleep stage scoring was done at 30-second intervals by experienced technicians according to the standard criteria [23]. Obstructive apnea was defined as a cessation of airflow for at least 10 seconds with the subject making an effort to breathe during apnea. Obstructive hypopnea was defined as an abnormal respiratory event with at least a 30% reduction in thoraco-abdominal movement or airflow as compared to baseline, lasting for at least 10 seconds, with a greater than 4% oxygen desaturation. The AHI was defined as the total number of apneas and hypopneas per hour of electroencephalographic sleep. An AHI of at least five events per hour of sleep established the diagnosis of OSA (primary snoring, AHI <5; mild OSA, AHI = 5.0–14.9; moderate OSA, AHI = 15.0–29.9; severe OSA, AHI≥30.0). The CPAP-treated patients had undergone a CPAP titration study with a manually titrated machine (GoodKnight 420E, Nellcor Puritan Bennett, California, U.S.A.) to get an optimal pressure before starting their treatment with either fixed or auto-adjusted positive airway pressure machines at home.

### Measurement of DNA methylation levels over the TLR2 promoter region and TLR6 gene body by bisulfite pyrosequencing method

Twenty milliliters of venous blood were withdrawn from healthy subjects and patients with severe OSA at around 07:30–08:30AM after overnight fast and sleep. The venous blood (20 ml) was also obtained before and after 6-month CPAP management in 7 patients with severe OSA who had been under CPAP therapy.

**Fig 1:**
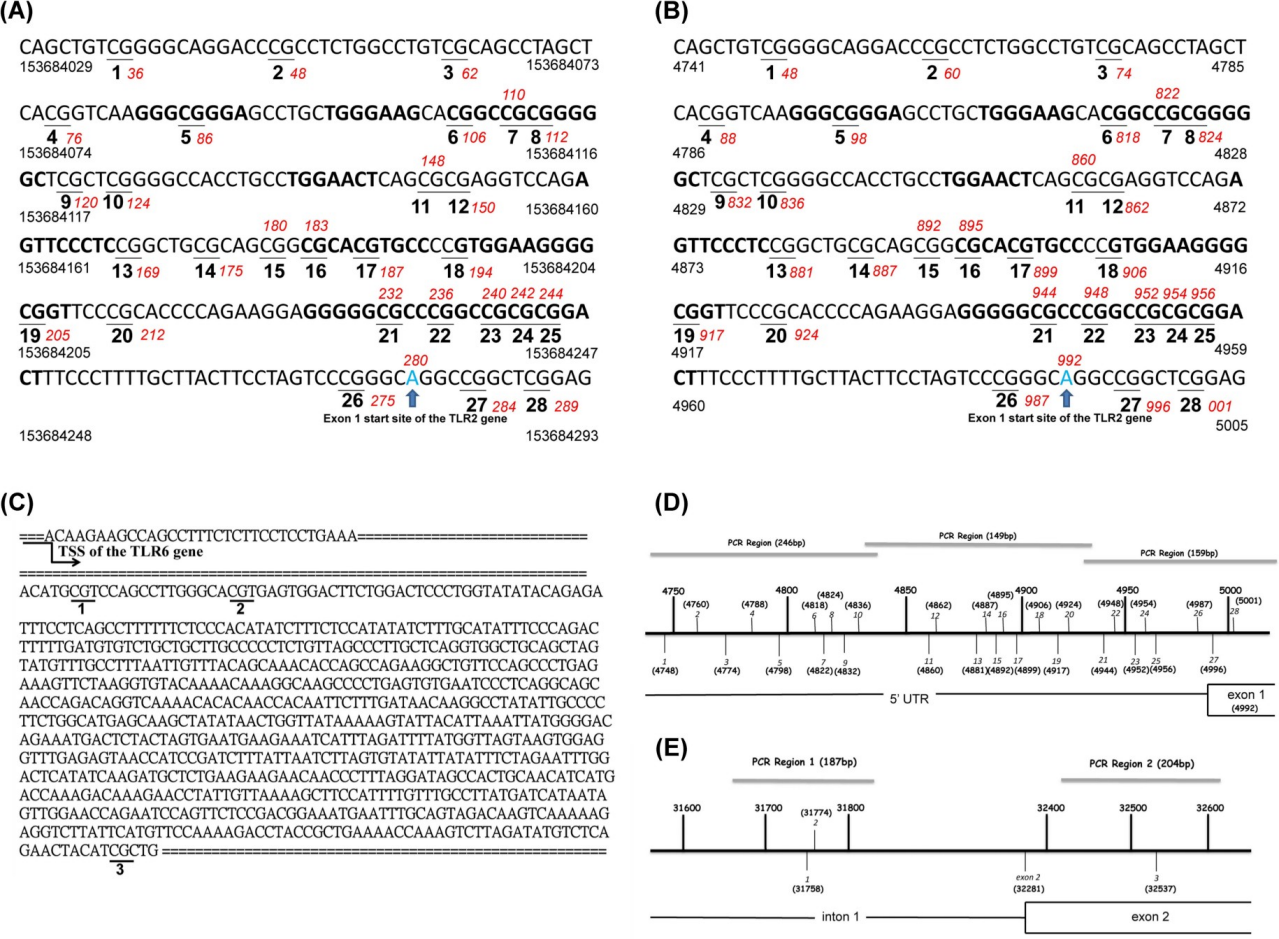
Diagrams showing CpG site locations of the *TLR2* and *TLR6* genes (genome build “GRCh38.p13”). (A)~(B): A zoom of the 28 CpG sites assayed in the *TLR2* gene and their genomic sequences based on NCBI Reference Sequence (NC_000004.12 and NG_016229.1). (C): A zoom of the 3 CpG sites assayed in the *TLR6* gene and their genomic sequences based on NCBI Reference Sequence (NG_028087.1). (C): Diagram showing CpG site locations of the *TLR2* gene. The CpG site #27 and #28 were in the exon 1 of the gene body, and the others were in the promoter regions. (D): Diagram showing CpG site locations of the *TLR6* gene. CpG site #1 and #2 were in the intron 1, and #3 was in the exon 2 of the gene body.

Genomic DNA was isolated from whole blood using a genomic DNA purification kit (Puregene). Three regions of the *TLR2* promoter element (NCBI Reference Sequence: NC_000004.12 and NG_016229.1), including 28 CpG sites, were amplified (Figs 1 and S1–S2). Two regions of the *TLR6* gene body, including 3 CpG sites (NCBI Reference Sequence: NG_028087.1) were analyzed (CpG site #1 and #2 were in the intron 1, and #3 was in the exon 2 of the gene body) (Figs 1 and S3–S4). Sodium bisulfate treatment was performed using EZ DNA Methylation^TM^ Kit (ZYMO RESEARCH, USA) and PCR amplification was performed using PyroMark PCR Kit (Qiagen, Germany). The PCR condition was 45 cycles of 95°C for 20 s, 50°C for 20 s, and 72°C for 20 s, followed by 72°C for 5 min. Primer sequences used for PCR amplification and pyrosequencing are listed in S1 Table. The biotin-labeled PCR product was captured by Streptavidin Sepharose^TM^ High Performance (GE Healthcare, Germany). Quantitation of cytosine methylation was done using the PyroMark Q24 system (Qiagen, Germany). The amount of C relative to the sum of the amounts of C and T at each CpG site was calculated as percentage. Representative pyrograms of CpG di-nucleotides assayed are presented in S5 Fig.

### Measurement of TLR2 and TLR6 total protein expression by enzyme-linked immunosorbent assay (ELISA)

TLR2 and TLR6 protein expression were measured by using TLR2 and TLR6 Human ELISA Protocol Kit (USCN Life Science Inc, USA)

### Statistical analysis

Data were expressed as the mean ± standard deviation. Independent samples *t*-test or Mann–Whitney U test was used for comparing continuous variables. Categorical variables were analyzed using Chi-square test. ANOVA model followed by post-hoc Bonferroni analysis was performed to analyze the differences in continuous variables among HS, non-EDS and EDS groups. Paired samples *t*-test was used to compare the change in the levels of DNA methylation and protein expressions before and after CPAP management. Multivariate linear regression with hierarchical comparison was performed in two steps with all potential co-variables (age, body mass index<BMI>, gender, diabetes mellitus<DM>, hypertension, stroke, valvular heart disease <VHD>, ischemic heart disease <IHD>, congestive heart failure<CHF>, arrhythmia, rhinitis, obstructive airway disease<OAD>, malignancy, renal failure and gastroesophageal reflux disease<GERD> *were entered in the first step*, and the OSA or EDS *were entered in the second step*) to determine independent factors contributing to the DNA methylation of the *TLR2* promoter region and the *TLR6* gene body, and their protein expressions in patients. A *p* value of less than 0.05 was considered statistically significant. All statistical analyses were performed with SPSS for Windows, version 18.0 (SPSS, Chicago, IL, USA). To assist in the interpretation of p-values given the number of statistical tests performed, q-values (false discovery rate) were calculated separately for multiple comparisons of the DNA methylation and protein expression levels by Benjamini-Hochberg test using R Console software, version 3.4.0. (2017 The R Foundation for Statistical Computing). A *q* value threshold of 0.1 was selected to separate false from true discoveries, so up to 10% of declared discoveries should be expected to be false.

## Results

### Demography

A total of 18 healthy subjects (HS) and 58 patients with severe OSA were included in the study. Patients with OSA were older (37.72±9.20 vs. 49.43±10.07, p < .001) and more obese (BMI 24.54±3.58 vs. 29.06±5.23, p = .001). Male gender and prevalent hypertension were also more frequently noted in patients with severe OSA (Table 1).

**Table 1:** Demographic characteristics between healthy subjects and patients with severe OSA.

	HS(n = 18)	OSA (n = 58)	*p* value
**Age**	37.72±9.20	49.43±10.07	< .001
**BMI**	24.54±3.58	29.06±5.23	.001
**Gender(M/F)**	10(55.6%)/8(44.4%)	54(93.1%)/6(10.3%)	.001
**DM**	0(0%)	8(13.8%)	.187
**Hypertension**	0(0%)	22(37.9%)	.002
**Stroke**	0(0%)	1(1.7%)	1.00
**VHD**	1(5.6%)	10(17.2%)	.441
**IHD**	0(0%)	3(5.1%)	1.00
**CHF**	0(0%)	2(3.4%)	1.00
**Arrhythmia**	0(0%)	1(1.7%)	1.00
**Rhinitis**	3(16.7%)	13(22.4%)	.75
**OAD**	0(0%)	7(12.1%)	.192
**Malignancy**	0(0%)	4(6.9%)	.57
**Renal failure**	2(11.1%)	6(10.3%)	1.00
**GERD**	1(5.6%)	15(25.9%)	.10
**WBC**	6.32±1.52	6.84±1.99	.351
**TSH**	1.44±.73	1.88±1.40	.30
**Chol**	187.93±33.36	190.96±35.01	.76
**TG**	111.80±58.65	165.45±109.11	.07
**HbA1c**	5.39±.31	8.96±16.76	.48

### DNA methylation and protein expression levels in OSA and healthy subjects

**Fig 2:**
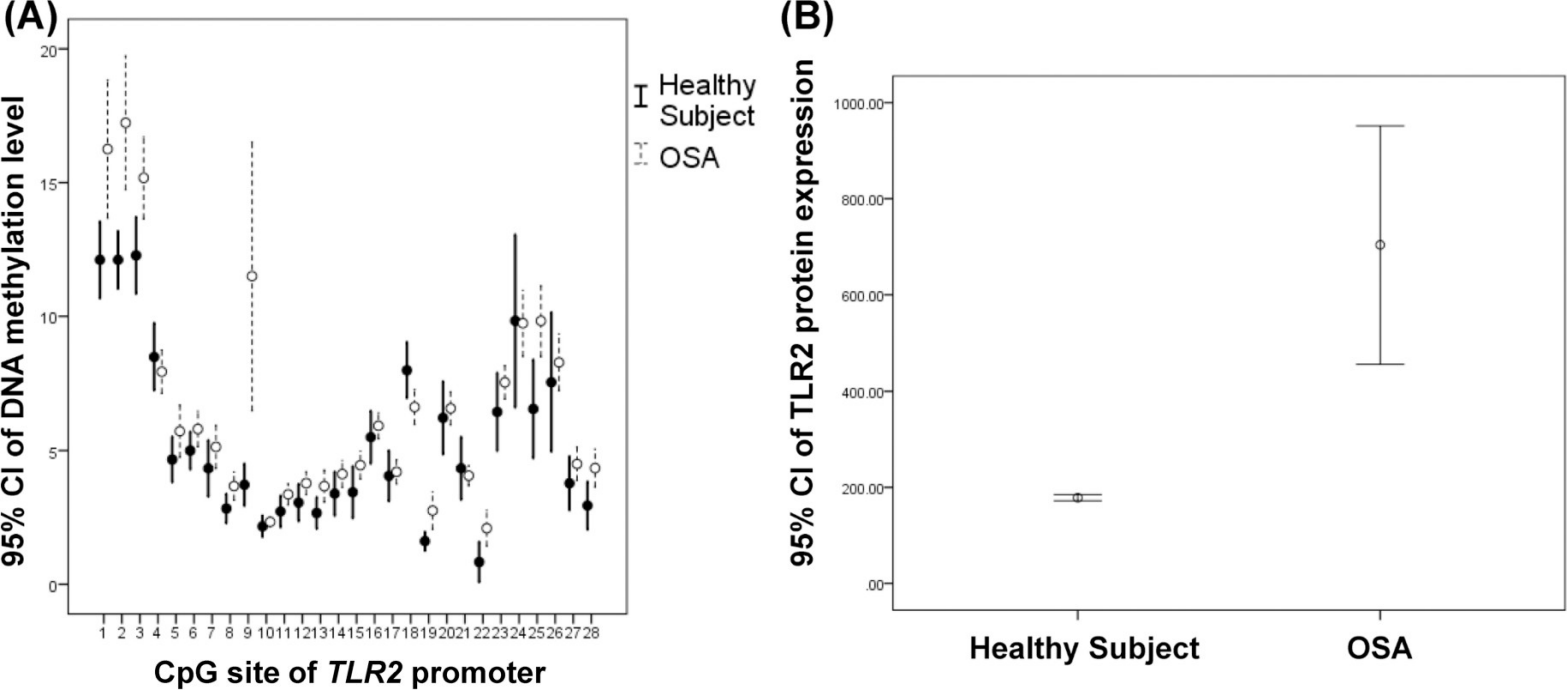
Comparisons of DNA methylation levels over the *TLR2* promoter region and TLR2 protein expressions. (A)DNA methylation levels over CpG site #1, #2, #3, #8, #9, #13, #19, #22, #25 and #28 of the *TLR2* promoter region were increased (*p* = .006, < .001, .006, .027, .003, .018, .004, .013, .005, and .015 respectively ), while DNA methylation levels over CpG site #18 of the *TLR2* promoter region was decreased (*p* = .027). (B)TLR2 protein expression were increased in patients with severe OSA (*p* < .001).

**Fig 3:**
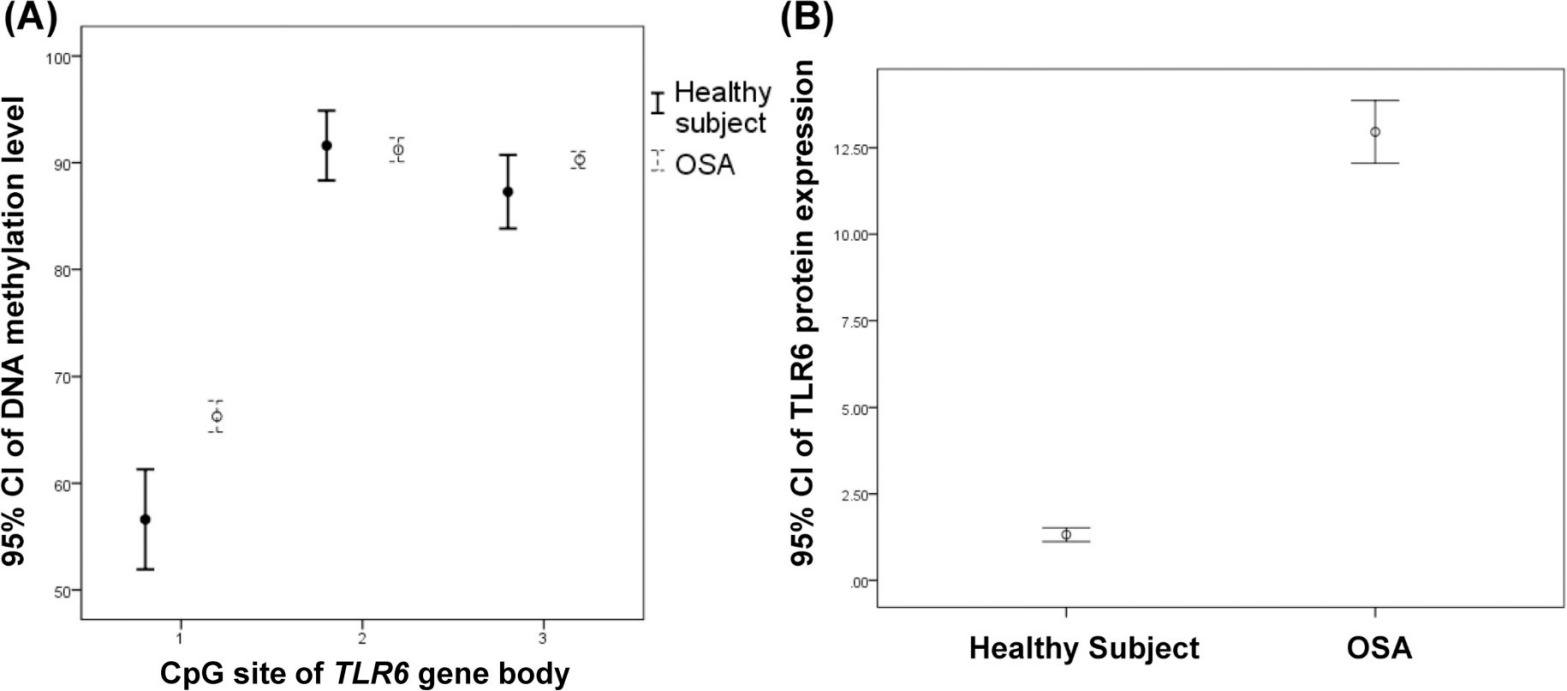
Comparisons of DNA methylation levels of the *TLR6* gene body and TLR6 protein expression. (A)DNA methylation levels over CpG site #1 of *TLR6* gene body and (B)TLR6 protein expressions were increased in patients with severe OSA (*p* < .001 and < .001 respectively).

DNA methylation levels over CpG sites #1, #2, #3, #8, #9, #13, #19, #22, #25 and #28 of the *TLR2* promoter region and TLR2 protein expression were all increased in patients with severe OSA as compared with HS, while DNA methylation levels over CpG site #18 of the *TLR2* promoter region was decreased (Fig 2 and S2 Table). Both DNA methylation levels over CpG site #1 of the *TLR6* gene body and TLR6 protein expressions were significantly increased in patients with severe OSA versus HS (Fig 3 and S2 Table).

Multivariate linear regression analysis showed that OSA was the independent factor of DNA methylation levels over CpG sites #1, #2, #3, #18, #25 and #28 of the *TLR2* promoter region, CpG sites #1 and #3 of the *TLR6* gene body, and protein expressions of TLR6 (Tables 2 and S3). Apnea-Hypopnea index (AHI) or oxygen de-saturation index (ODI) were also independent factors of DNA methylation levels of the *TLR2* promoter region, *TLR6* gene body, and protein expressions of TLR6 (S4 and S5 Tables). These results remained statistically significant in DNA methylation levels over CpG sites #1 and #3 of the *TLR6* gene body after correction for multiple comparisons with all *q* values <0.1. (Tables 2 and S6–S8). In addition, the DNA methylation levels over CpG sites #1 and #3 were significant correlated to protein expressions of TLR6 after the analysis using Pearson correlation (p < .001 and p = .028 respectively).

**Table 2:** Summary of results of multivariate linear regression and multiple comparison: OSA is the independent risk factor of DNA methylation levels over CpG site #1, #2, #3, #18, #25 and #28 of the *TLR2* promoter region, CpG site #1 and #3 of *TLR6* gene body, and protein expression of TLR6. (The complete data was presented at S6 Table).

		Multivariate linear regression						Multiple comparisons
		*△F*	*p*	*△R*^*2*^	*β*	*t*	*pr*^*2*^	*q*
***TLR2* promoter region**	CpG#1	4.546	.037	.051	.327	2.132	.071	.153
	CpG#2	5.202	.026	.055	.339	2.281	.081	.123
	CpG#3	5.293	.025	.053	.335	2.301	.082	.123
	CpG#18	4.333	.042	.056	-.342	-2.082	.069	.154
	CpG#25	6.221	.015	.072	.389	2.494	.095	.123
	CpG#28	5.856	.019	.070	.383	2.420	.090	.123
***TLR6* gene body**	CpG#1	20.736	*<* .001	.192	.635	4.554	.260	< .001
	CpG#3	9.421	.003	.120	.502	3.069	.138	.033
**Protein expression**	TLR6	97.805	*<* .001	.325	.825	9.890	.645	< .001

### DNA methylation and protein expression levels in OSA with and without excessive daytime sleepiness

**Fig 4:**
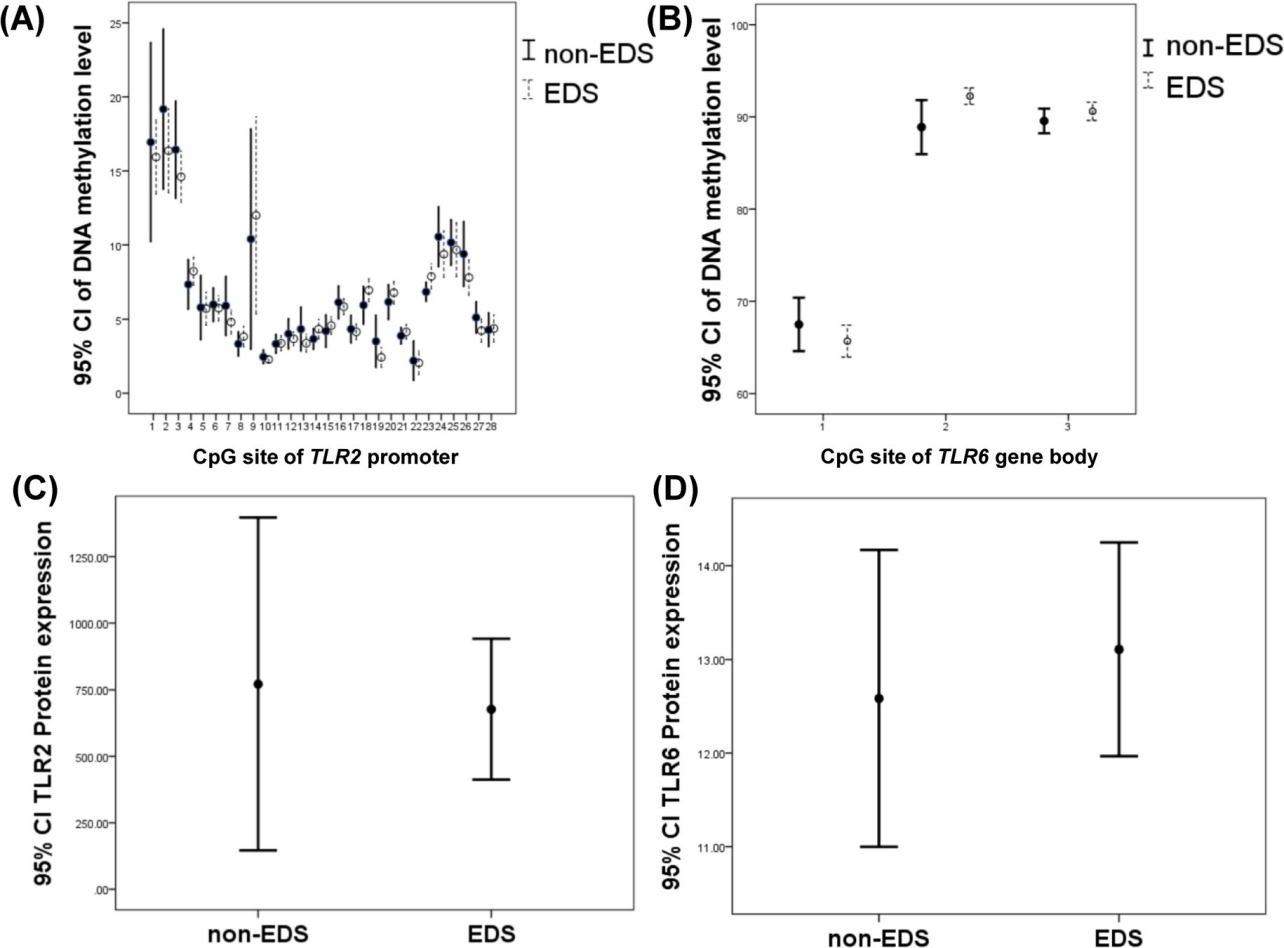
Comparisons of DNA methylation levels over the *TLR2* promoter region, *TLR6* gene body, and protein expressions of TLR2 and TLR6 between non-EDS and EDS groups. (A)~(D) The DNA methylation levels over the *TLR2* promoter region, and protein expressions of TLR2 and TLR6 were not significantly different except that CpG site #2 of the *TLR6* gene body was hypermethylated significantly in the EDS group (*p* = .032).

All the severe OSA patients were divided into two groups based on Epworth sleepiness scale (ESS): 18 patients with ESS less than or equal to 10 (non-EDS group) and 40 patients with ESS more than 10 (EDS group). Using ANOVA model followed by post-hoc Bonferroni correction analysis among the HS, non-EDS and EDS groups, we found that the demographic characteristics were not significantly different between non-EDS and EDS groups, but these two groups had significantly older age, were more obese and had a larger proportion of prevalent hypertension than did the HS group (Table 3). DNA hypermethylation over CpG site #2 of the *TLR6* gene body was noted in the EDS group versus non-EDS group (Fig 4 and S9 Table). EDS was the independent factor of the DNA methylation level over CpG site #2 of the *TLR6* gene body after multivariate linear regression analysis and multiple comparisons. (S10 and S11 Tables).

**Table 3:** Demographic characteristics between healthy subjects, non-EDS and EDS patients.

	HS(n = 18)	Non-EDS(n = 18)	EDS(n = 40)	*p* value
**Age**	37.72±9.20	49.16±7.79	49.56±11.06	< .001*
**BMI**	24.54±3.58	28.74±5.67	29.21±5.09	.004**
**Gender(M/F)**	10(55.6%)/8(44.4%)	14(77.8%)/4(22.2%)	38(95%)/2(5%)	.001^@^
**DM**	0(0%)	3(16.7%)	5(12.5%)	.247
**Hypertension**	0(0%)	7(38.9%)	15(37.5%)	.009^@@^
**Stroke**	0(0%)	0(0%)	1(2.5%)	.643
**VHD**	1(5.6%)	4(22.2%)	6(15%)	.406
**IHD**	0(0%)	0(0%)	3(7.5%)	.252
**CHF**	0(0%)	1(5.6%)	1(2.5%)	.607
**Arrhythmia**	0(0%)	1(5.6%)	0(0%)	.214
**Rhinitis**	3(16.7%)	4(22.2%)	9(22.5%)	.200
**OAD**	0(0%)	1(5.6%)	6(15%)	.161
**Malignancy**	0(0%)	3(16.7%)	1(2.5%)	.049
**Renal failure**	2(11.1%)	1(5.6%)	5(12.5%)	.714
**GERD**	1(5.6%)	3(16.7%)	12(30%)	.099
**WBC**	6.32±1.52	6.85±1.79	6.82±2.09	.648
**TSH**	1.44±.73	2.09±1.96	1.79±1.10	.441
**Chol**	187.93±33.36	196.10±38.45	188.39±33.40	.702
**TG**	111.80±58.65	141.62±78.89	177.36±120.63	.091
**HbA1c**	5.39±.31	10.91±20.99	8.07±14.64	.638

* *p* = .002 and *p* < .001were noted in HS versus Non-EDS and EDS group respectively; ** *p* = .035 and *p* = .004 were noted in HS versus Non-EDS and EDS group respectively;

^@^ *p* = .148 and *p* = .001 were noted in HS versus Non-EDS and EDS group respectively;

^@@^ *p* = .034 and *p* = .011 were noted in HS versus Non-EDS and EDS group respectively.

### DNA methylation and protein expression levels after CPAP management

**Fig 5:**
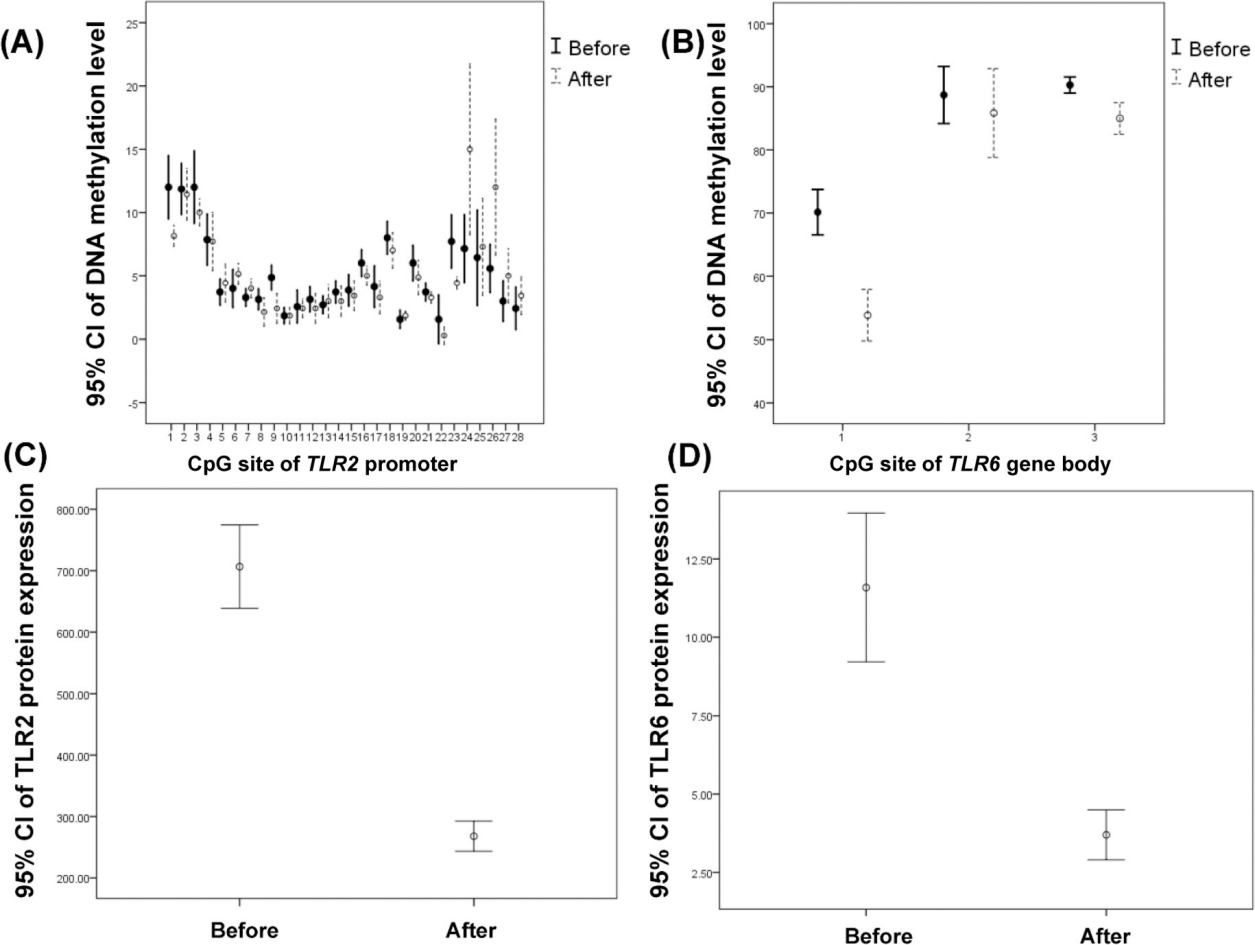
Changes in DNA methylation and protein expression levels of the *TLR2* promoter region and *TLR*6 gene body in 7 selected severe OSA patients before and after more than 6-month CPAP treatment. (A) DNA methylation levels over CpG site #1, #9 and #23 of *TLR2* promoter region were decreased (*p* = .018, .015 and .006 respectively) and DNA methylation levels over CpG site #24 and #26 of *TLR2* promoter region were increased (*p* = .028 and .042 respectively). (B)~(D) DNA methylation levels over CpG sites #1 and #3 of the *TLR6* gene body, and TLR2 and TLR6 protein expressions were reduced (*p* < .001, *p* = .003, *p* < .001 and < .001 respectively).

In seven patients with severe OSA who had received more than 6 months of CPAP treatment, DNA methylation levels over CpG sites #1, #9 and #23 of the *TLR2* promoter region, CpG sites #1 and #3 of the *TLR6* gene body, and TLR2 and TLR6 protein expressions were all reduced, and DNA methylation levels over CpG sites #24 and #26 of the *TLR2* promoter region were both elevated (Fig 5 and S12 Table). After multiple comparison corrections with the Benjamini and Hochberg method, CPAP management was still an independent factor for the changes in the DNA methylation levels of CpG sites #1, #9, and #23 of the *TLR2* promoter region, and CpG sites #1 and #3 of the *TLR6* gene body (S13 Table)

## Discussion

The main findings of our study are summarized as follows(Tables 4 and 5):

1. Both DNA methylation levels of the *TLR6* gene body over CpG site #1 and #3, and protein expressions of TLR6 were significant correlated, and increased in patients with severe OSA.
2. Aberrant DNA methylation levels of the *TLR2* promoter region, hypermethylated *TLR6* gene body, and increased protein expressions of TLR6 were all independently associated with higher AHI and ODI.
3. DNA methylation levels of the *TLR6* gene body over CpG site #2 were increased in OSA with EDS phenotype.
4. The altered DNA methylation levels over CpG sites #1 and #3 of the *TLR6* gene body as well as TLR2 and TLR6 protein expressions were reversed after nasal CPAP management.

These results provide evidence for the first time that aberrant DNA methylation of the *TLR2* promoter region and *TLR6* gene body might be involved in the consequence of OSA and its clinical phenotypes.

**Table 4:** The summarized results of multivariate linear regression and multiple comparisons in DNA methylation levels of *TLR2* promoter region, *TLR6* gene body, and protein expressions of TLR2 and TLR6 between healthy subjects and patients with severe OSA. The DNA methylation levels over CpG#1, #2, #3, #18, #25, and #28 of *TLR2*, and CpG#1 and CpG#3 of *TLR6* were significant differently between HS and OSA after multivariate linear regression. However, only the CpG#1 and CpG#3 of *TLR6* were increased significantly in OSA after multiple comparisons. The TLR6 expression was also increased significantly in OSA.

			HS (n = 18)	OSA(n = 58)	*p*	*q*
*TLR2* promoter region	CpG#1	cg153684036	12.11±2.888	16.25±9.822	.037	0.1526250
	CpG#2	cg153684048	12.11±2.166	17.24±9.528	.026	0.1225714
	CpG#3	cg153684062	12.28±2.886	15.18±5.823	.025	0.1225714
	CpG#18	cg153684194	8.00±2.086	6.63±2.492	.042	0.1540000
	CpG#25	cg153684244	6.56±3.698	9.83±5.016	.015	0.1225714
	CpG#28	cg153688942	2.94±1.798	4.34±2.686	.019	0.1225714
*TLR6* gene body	CpG#1	cg13006575	56.61±9.432	66.26±5.565	*<* .001	0.0004455
	CpG#3	cg25769980	87.28±6.952	90.28±2.996	.003	0.0330000
Protein expression	TLR6		1.3219±.40024	12.9591±3.29140	*<* .001	na

**Table 5:** The summarized results of multivariate linear regression and multiple comparisons in DNA methylation levels of *TLR2* promoter region, *TLR6* gene body, and protein expressions of TLR2 and TLR6 in OSA before and after CPAP management. (genome build “GRCh38.p13”). The DNA methylation levels over CpG#1, #9, #23, #24, and #26 of *TLR2*, and CpG#1 and CpG#3 of *TLR6* were significant differently in multivariate linear regression. After CPAP management, only the CpG#1 and CpG#3 of *TLR6* were decreased significantly in multiple comparisons. The TLR2 and TLR6 expression was also decreased significantly after CPAP management.

			Before	After	Difference	*p*	*q*
*TLR2* promoter region	CpG#1	cg153684036	12.00±2.70	8.142±.89	3.85±3.18	.018	0.08485714
	CpG#9	cg153684120	4.86±1.06	2.42±1.27	2.42±1.90	.015	0.08250000
	CpG#23	cg153684240	7.71±2.28	4.42±.53	3.28±2.05	.006	0.03960000
	CpG#24	cg153684242	7.14±2.91	15.00±7.30	-7.85±7.22	.028	0.11550000
	CpG#26	cg153684275	5.57±2.07	12.00±5.83	-6.42±6.60	.042	0.15400000
*TLR6* gene body	CpG#1	cg13006575	70.14±3.89	53.86±4.38	16.29±3.45	< .001	0.00026400
	CpG#3	cg25769980	90.29±1.38	85.00±2.71	5.29±2.93	.003	0.02475000
Protein expression	TLR2		706.53±73.29	268.04±26.62	438.49±69.84	< .001	na
	TLR6		11.59±2.57	3.70±.86	7.89±1.84	< .001	na

Persistent hypoxia can induce hypermethylation or demethylation of specific genes [24–26], while the impact of chronic intermittent hypoxia on DNA methylation remains uncertain. Previous studies [15, 27] have found aberrant DNA methylations in several genes and might constitute an important determinant of disease severity and vulnerability to EDS in OSA. In addition, both TLR2/6 co-expressions on neutrophil and monocyte were increased either in OSA patients or with intermittent hypoxia with re-oxygenation treatment in vitro [16]. The cause-and-effect relationship between hypermethylation or demethylation of these genes and the consequences of OSA are still unclear, and we speculated that chronic intermittent hypoxia might contribute to systemic inflammation and adverse consequences through inducing aberrant DNA methylation. However, it is equally possible that these epigenetic changes might occur in the prenatal period or early life and predispose subjects to an epigenotype and subsequently a phenotype with more frequent hypoxic events during sleep in adulthood.

Toll-like receptors play central roles in the innate immune response by recognizing conserved structural patterns in diverse microbial molecules [28]. They are key mediators of innate immunity in both vertebrates and invertebrates, respond to various pathogen-associated stimuli, and transduce the complex signaling responses that are required for inflammation and for the subsequent development of adaptive immunity [29]. TLR2 has been shown to sense bacterial lipopeptides: it heterodimerizes either with TLR1 to recognize tri-acylated lipopeptides or with TLR6 to recognize di-acylated lipopeptides [30]. Intermittent hypoxia induces several inflammatory cascades, including the production of reactive O2 species, HIF-1 activation, and activated TLRs [3].

Our results emphasize the role of TLR6 in association with its epigenetic change in OSA. Aberrant DNA methylation levels of the *TLR2* promoter region and *TLR6* gene body as well as protein expressions of TLR6 were all independently associated with AHI and ODI. The reversion of the DNA methylation levels of *the TLR2* promoter region, *TLR6* gene body and protein expression of TLR2/TLR6 after CPAP management were also observed in seven selected patients. These results suggest that chronic intermittent hypoxia during sleep would induce the changes in the epigenesis of *TLR2/TLR6* genes and the downstream protein expressions, especially TLR6. TLR6 might well serve as a biomarker in the diagnosis, disease severity, or evaluation of management in serial follow-up in OSA, but further investigation is warranted.

Although DNA methylation levels over several CpG sites in the *TLR2* promoter region were increased in OSA patients, hypomethylated CpG site #18 in association with increased TLR2 protein expression was noted. In our previous study [20], we showed hyper-methylated CpG site #18 was in association with decreased TLR protein expression in active pulmonary TB patients. Thus, we speculated that CpG#18 methylation status may have a major effect on *TLR2* gene expression under chronic intermittent hypoxic stimuli in OSA. Given the hypermethylated CpG sites #1 and #3 of the *TLR6* gene body are in association with increased TLR6 protein expressions, we speculated that heterodimerization between TLR2 and TLR6 might be enhanced through aberrant DNA methylation of these two genes as well as through hypermethylation of the *AKT1* and *LY86* genes, both of which are involved in the activation of TLR signaling [31–34].

EDS is one of the prominent symptoms of OSA [35], and in particular, it is known to be a predisposing factor for accidents, interpersonal (*communicative*) problems, and reduced productivity [36–38]. EDS is also correlated with the severity of OSA [39] and might be a useful clinical marker to identify patients at risk of metabolic syndrome, hypertension and low-grade inflammation [40–42], which might all be linked to cardiometabolic morbidity and mortality. Nocturnal hypoxemia is a major determinant of EDS in Chinese OSA patients [39]. In our patients, hypertension was more prevalent in OSA but not significantly different between non-EDS and EDS groups. Increased DNA methylation over CpG site #2 of the *TLR6* gene body was noted in the EDS group. This finding suggests that hypermethylation of the *TLR6* gene body might also participate in the development of the EDS phenotype, but further investigation in a larger cohort of patients is necessary.

The limitations of our study should be acknowledged. Firstly, we enrolled only patients with severe OSA in order to avoid the potential confounding variables emanating from low severity of OSA. Secondly, this was a small population and cross-sectional case-control study, but we had direct comparison before and after CPAP treatment in some selected OSA patients. Further studies with sufficiently large sample sizes are required for internal and external validity and reliability of the results. Thirdly, although the difference of DNA methylation level between HS and OSA patients in statistically, it is insufficient for indicating the regulation of *TLR2* and *TLR6* expression. The further investigation by performing luciferase assay should be considered. Fourthly, the mechanisms of regulation of gene expression by DNA methylation of promoter region are thought that highly methylated DNA region inhibit for binding of transcription factor to their binding motif. However, we did not check what of the region of *TLR2* promoter highly methylated in patient contains transcription factor binding motif. Fifthly, to assess the compliance and adherence of CPAP management by using CPAP >4 hours per night for 6 months might be insufficient. Further reassessment of compliance and adherence of CPAP management by using residual AHI (eg, "effective AHI [43]) is warranted. Sixthly, further *in vitro* study is needed to establish the cause-and-effect relationship between intermittent hypoxia and aberrant DNA methylation of the *TLR2* and *TLR6* genes. Seventhly, these particular methylation may have biological significance but additional support from epigenetic atlases and/or multiple site tests should be warranted.

## Conclusions

This study provides a novel finding of aberrant DNA methylation in OSA. Hyper-methylated *TLR6* gene bodies are associated with the consequence of severe OSA and its EDS phenotype. Increased protein expressions of TLR6 might serve as a biomarker for OSA. CPAP management partly reversed the altered DNA methylation levels and protein expressions of TLR2 and TLR6. Further verification and investigation of underlying mechanisms are warranted.

## Supporting information

S1 Fig Graphic view of of *TLR2* gene from NCBI BLAST. (TIF)

S2 Fig Graphic view of of *TLR2* gene from NCBI BLAST. (TIF)

S3 Fig Graphic view of of *TLR6* gene from NCBI BLAST. (TIF)

S4 Fig Graphic view of of *TLR6* gene from NCBI BLAST. (TIF)

S5 Fig The pyrograms of the TLR2 and TLR6 genes: A representative pyrogram showing the percentage of methylation at CpG sites of TLR2 gene (A~C) and TLR6 gene (D~E) in a patient with severe OSA.
(TIF)

S1 Table Primers of PCR amplification and pyrosequencing in measuring DNA methylation of the *TLR2* promoter region and *TLR6* gene body. (DOCX)

S2 Table DNA methylation levels of *TLR2* promoter region, *TLR6* gene body, and protein expressions of TLR2 and TLR6 between HS and patients with severe OSA. (DOCX)

S3 Table Multivariate linear regression with hierarchical comparisons showed that OSA is the independent risk factor of DNA methylation levels over CpG site #1, #2, #3, #18, #25 and #28 of the *TLR2* promoter region, CpG site #1 and #3 of *TLR6* gene body, and protein expression of TLR6. (DOCX)

S4 Table Multivariate linear regression with hierarchical comparisons showed that AHI is the independent risk factor of DNA methylation levels over CpG site #1, #2, #3, #11, #12, #13, #15, #19, and #20 of the *TLR2* promoter, CpG site #1 of *TLR6* gene body, and protein expressions of TLR6. (DOCX)

S5 Table Multivariate linear regression with hierarchical comparisons showed that ODI is the independent risk factor of DNA methylation levels over CpG site #1, #2, #3, #8, #11, #12, #13, #15, #17, #19, and #22 of the *TLR2* promoter, CpG site #1 of *TLR6* gene body, and protein expressions of TLR6. (DOCX)

S6 Table Multiple comparisons of DNA methylation levels in OSA. A *q* value threshold of 0.1 was selected to separate false from true discoveries, and the first 2 would be significant.
(DOCX)

S7 Table Multiple comparisons of DNA methylation levels in AHI. A *q* value threshold of 0.1 was selected to separate false from true discoveries, and the first 7 would be significant.
(DOCX)

S8 Table Multiple comparisons of DNA methylation levels in ODI. A *q* value threshold of 0.1 was selected to separate false from true discoveries, and the first 11 would be significant.
(DOCX)

S9 Table DNA methylation levels of *TLR2* promoter region, *TLR6* gene body, and protein expressions of TLR2 and TLR6 in OSA without and with EDS. (DOCX)

S10 Table Multivariate linear regression with hierarchical comparisons showed that EDS is the independent risk factor of DNA methylation levels over CpG site #2 of *TLR6* gene body. (DOCX)

S11 Table Multiple comparisons of DNA methylation levels in EDS. A *q* value threshold of 0.1 was selected to separate false from true discoveries.
(DOCX)

S12 Table Difference of DNA methylation levels in OSA before and after CPAP management. (DOCX)

S13 Table Multiple comparisons of DNA methylation levels in CPAP management. A *q* value threshold of 0.1 was selected to separate false from true discoveries, and the first 5 would be significant.
(DOCX)

## Abbreviations

OSA: Obstructive sleep apnea
TLR: Toll-like receptors
EDS: Excessive daytime sleepiness
IH: Intermittent hypoxia
IHD: Ischemic heart disease
CHF: Congestive heart failure
CpG: Guanine dinucleotide sequence
IL1R2: Interleukin 1 receptor 2
AR: Androgen receptor
NPR2: Natriuretic peptide receptor 2
SP140: Speckled protein 140
CPAP: Continuous positive airway pressure
HIF: Hypoxia inducible factor
TB: Tuberculosis
HS: Healthy subjects
BMI: Body mass index
DM: Diabetes mellitus
VHD: Valvular heart disease
OAD: Obstructive airway disease
GERDm: Gastroesophageal reflux disease
AHI: Apnea-Hypopnea index
ODI: Oxygen de-saturation index
ESS: Epworth sleepiness scale
na: not available
