# Supplementary figures and images for: Aberrant DNA methylation of the toll-like receptors 2 and 6 genes in patients with obstructive sleep apnea
Source: PLoS One. 2020 Feb 18;15(2):e0228958. doi: 10.1371/journal.pone.0228958 (PMC7028278; doi:10.1371/journal.pone.0228958)

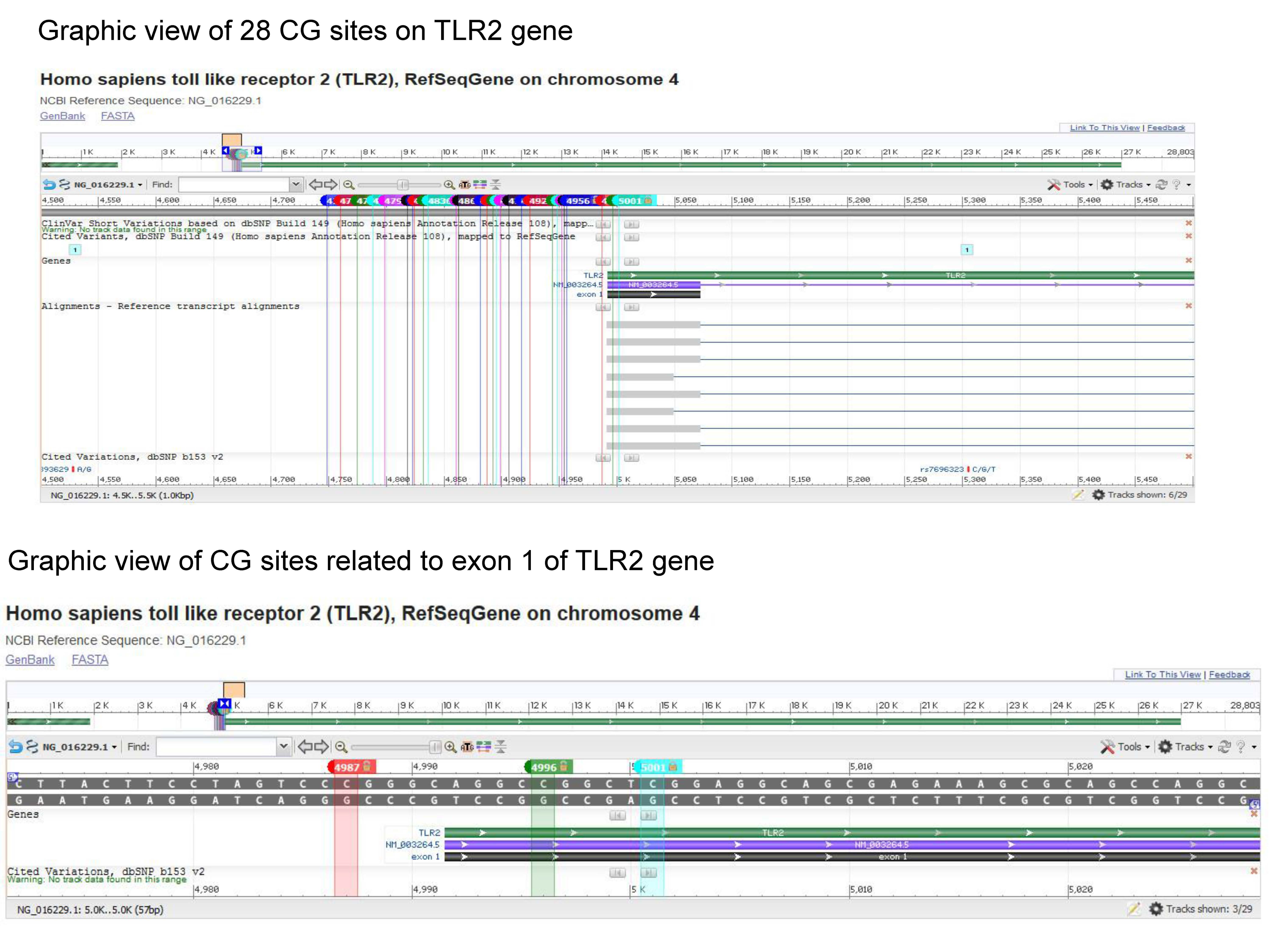

Supplement: S1 Fig — (TIF) [file pone.0228958.s001.tif]

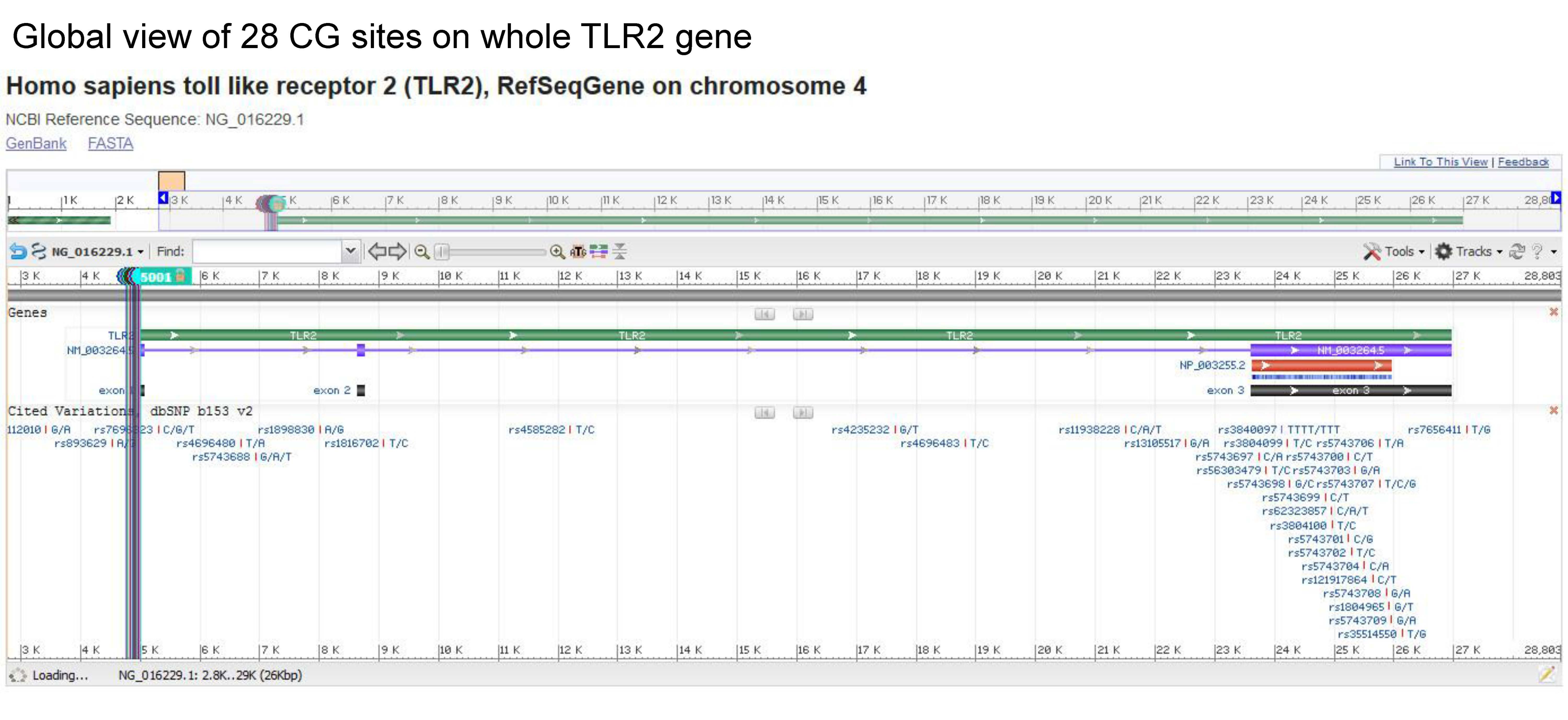

Supplement: S2 Fig — (TIF) [file pone.0228958.s002.tif]

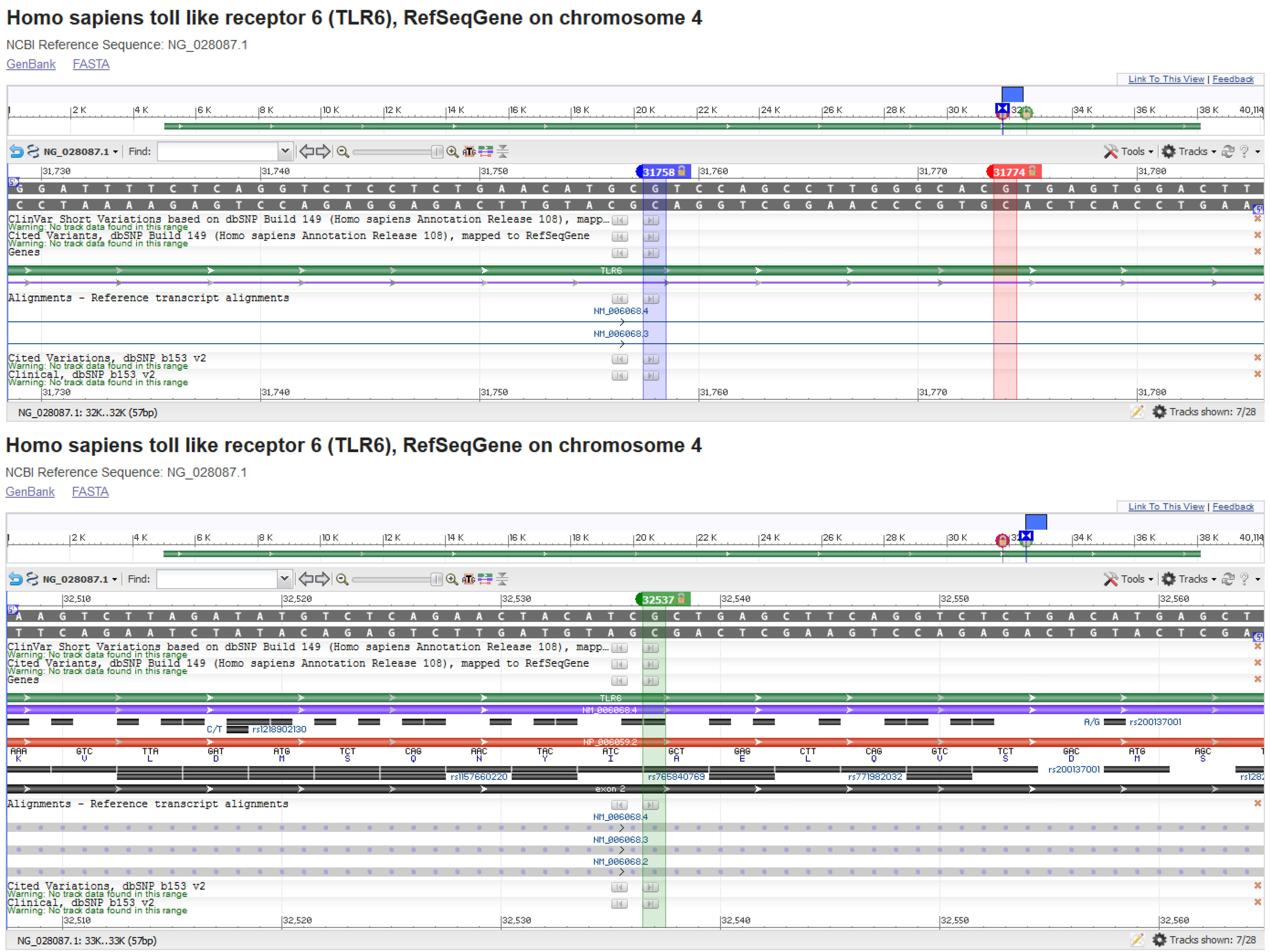

Supplement: S3 Fig — (TIF) [file pone.0228958.s003.tif]

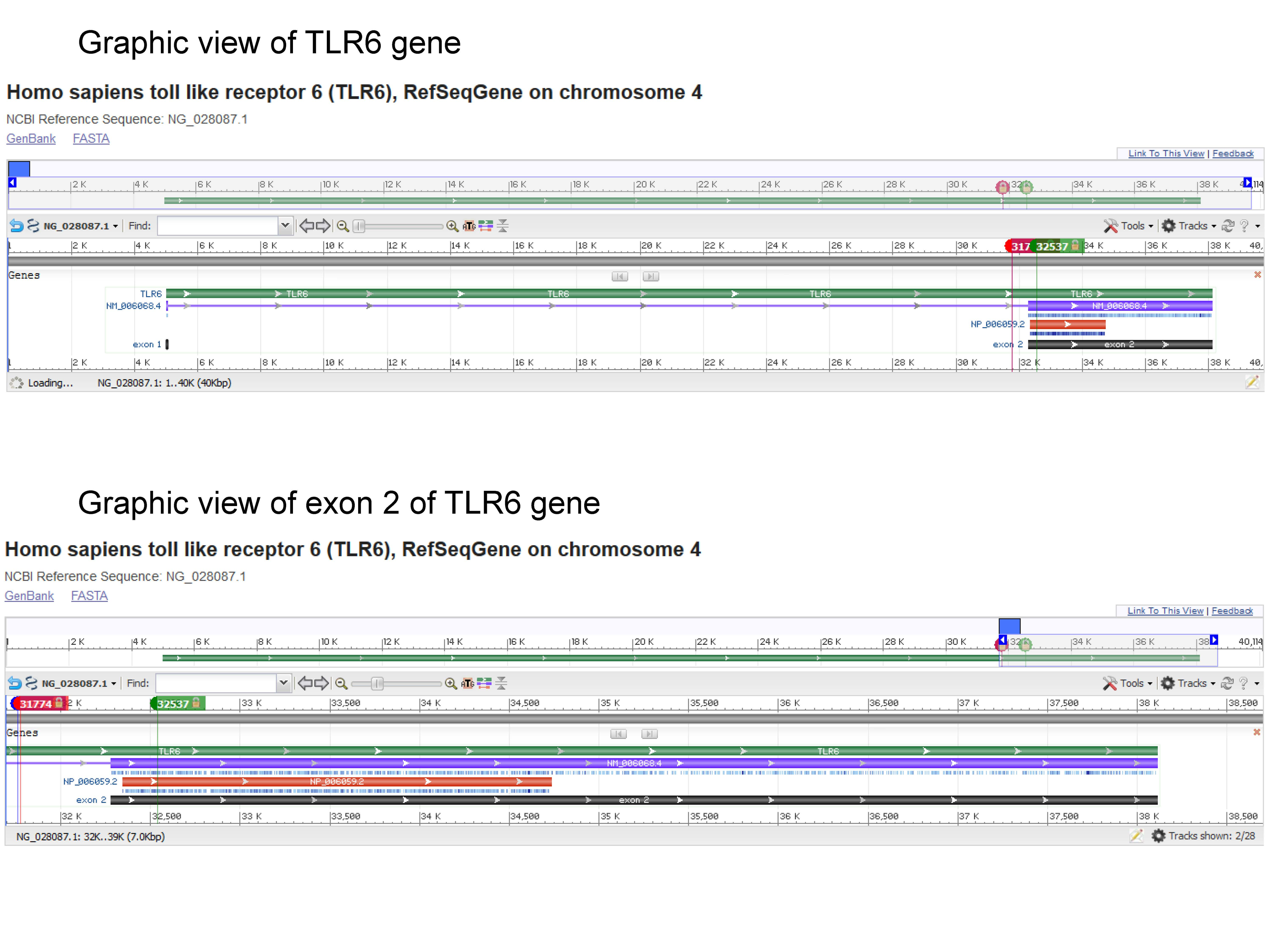

Supplement: S4 Fig — (TIF) [file pone.0228958.s004.tif]

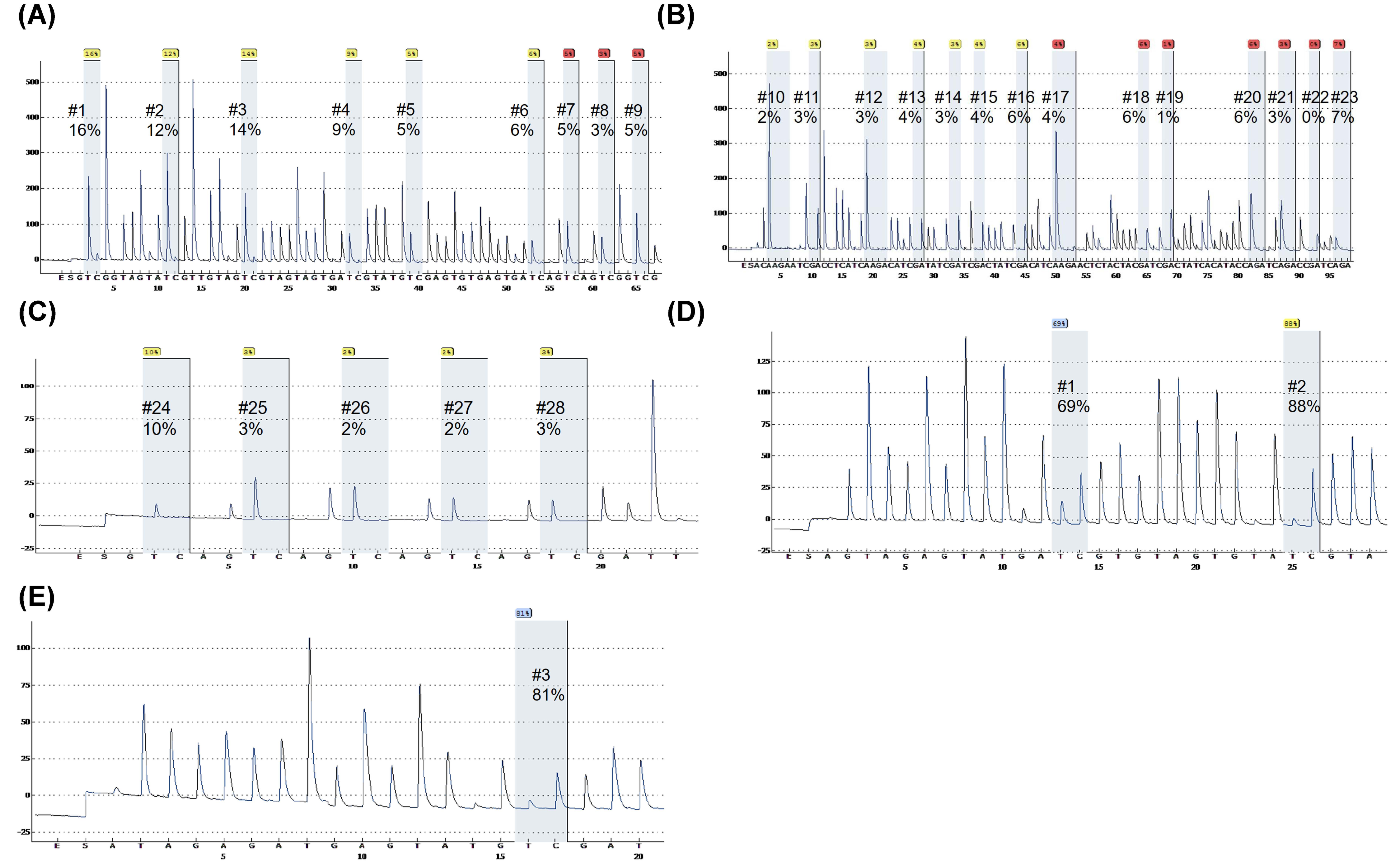

Supplement: S5 Fig — The pyrograms of the TLR2 and TLR6 genes: A representative pyrogram showing the percentage of methylation at CpG sites of TLR2 gene (A~C) and TLR6 gene (D~E) in a patient with severe OSA. (TIF) [file pone.0228958.s005.tif]
