# Supplementary material for: Aberrant DNA methylation of the toll-like receptors 2 and 6 genes in patients with obstructive sleep apnea
Source: PLoS One. 2020 Feb 18;15(2):e0228958. doi: 10.1371/journal.pone.0228958 (PMC7028278; doi:10.1371/journal.pone.0228958)
Supplement: S1 Table — (DOCX) [file pone.0228958.s006.docx]

**S1 Table. Primers of PCR amplification and pyrosequencing in measuring DNA methylation of the *TLR2* promoter region and *TLR6* gene body**.

| Primers | Sequences | |
| --- | --- | --- |
| *TLR2* |  | |
| Region 1 |  | |
| Forward PCR Primer | GGTATTTAGTTTTTTTTGTGGTTGTTAAT | |
| Reverse PCR Primer | Biotin-CTAAATTCCAAACAAATAACCC | |
| Forward Sequencing Primer | AGTTTTGTAAGGGGTAGTTG | |
| Region 2 |  | |
| Forward PCR Primer | Biotin-GGGAGTTTGTTGGGAAGT | |
| Reverse PCR Primer | AACCCCCCCTCCTTCTAAAAT | |
| Reverse Sequencing Primer | CCCCCTCCTTCTAAAATA | |
| Region 3 |  | |
| Forward PCR Primer | GGGGTTATTTGTTTGGAAT | |
| Reverse PCR Primer | Biotin-ACCTACCCCAAACTAAAAAATAAACAAAA | |
| Forward Sequencing Primer | GTATTTTAGAAGGAGGGGG | |
| *TLR6* |  |  |
| Region 1 |  |  |
| Forward PCR Primer | GAGAAAAAAGGTTGAGGAAATTTTTGTATA |  |
| Reverse PCR Primer | Biotin-TACTTAACCAATCTATTCACAACTCTAC |  |
| Forward Sequencing Primer | AGGAAATTTTTGTATATATTAGGGA |  |
| Region 2 |  |  |
| Forward PCR Primer | GATTTTGTTATGGGAAAGTTTTAAAATTGT |  |
| Reverse PCR Primer | Biotin-ATAATTAAAACCAAAATCCAATTCTCC |  |
| Forward Sequencing Primer | TGTTAATTTTGATAGAAAGTTTATG |  |
|  |  |  |
|  |  |  |
|  |  |  |
|  |  |  |
