# Supplementary material for: Aberrant DNA methylation of the toll-like receptors 2 and 6 genes in patients with obstructive sleep apnea
Source: PLoS One. 2020 Feb 18;15(2):e0228958. doi: 10.1371/journal.pone.0228958 (PMC7028278; doi:10.1371/journal.pone.0228958)
Supplement: S2 Table — (DOCX) [file pone.0228958.s007.docx]

**S2 Table. DNA methylation levels of *TLR2* promoter region, *TLR6* gene body, and protein expressions of TLR2 and TLR6 between healthy subjects(HS) and patients with severe OSA. (**genome build “GRCh38.p13”)

|  |  |  | HS (n=18) | OSA(n=58) | *p* |
| --- | --- | --- | --- | --- | --- |
| *TLR2* promoter region | CpG#1 | cg153684036 | 12.11±2.888 | 16.25±9.822 | .006 |
|  | CpG#2 | cg153684048 | 12.11±2.166 | 17.24±9.528 | <.001 |
|  | CpG#3 | cg153684062 | 12.28±2.886 | 15.18±5.823 | .006 |
|  | CpG#4 | cg153684076 | 8.50±2.503 | 7.95±3.075 | .491 |
|  | CpG#5 | cg153684086 | 4.67±1.715 | 5.72±3.717 | .248 |
|  | CpG#6 | cg153684106 | 5.00±1.414 | 5.81±2.523 | .092 |
|  | CpG#7 | cg153684110 | 4.33±2.114 | 5.14±3.075 | .304 |
|  | CpG#8 | cg153684112 | 2.83±1.098 | 3.67±2.012 | .027 |
|  | CpG#9 | cg153684120 | 3.72±1.565 | 11.50±19.072 | .003 |
|  | CpG#10 | cg153684124 | 2.17±.786 | 2.33±.726 | .405 |
|  | CpG#11 | cg153684148 | 2.72±1.179 | 3.36±1.495 | .101 |
|  | CpG#12 | cg153684150 | 3.06±1.392 | 3.78±1.644 | .095 |
|  | CpG#13 | cg153684169 | 2.67±1.188 | 3.67±2.294 | .018 |
|  | CpG#14 | cg153684175 | 3.39±1.650 | 4.12±1.908 | .146 |
|  | CpG#15 | cg153684180 | 3.44±1.947 | 4.45±1.968 | .061 |
|  | CpG#16 | cg153684183 | 5.50±1.978 | 5.93±1.850 | .403 |
|  | CpG#17 | cg153684187 | 4.06±1.893 | 4.20±1.735 | .761 |
|  | CpG#18 | cg153684194 | 8.00±2.086 | 6.63±2.492 | .027 |
|  | CpG#19 | cg153684205 | 1.61±.698 | 2.76±2.649 | .004 |
|  | CpG#20 | cg153684212 | 6.22±2.734 | 6.58±2.356 | .591 |
|  | CpG#21 | cg153684232 | 4.33±2.351 | 4.06±1.425 | .552 |
|  | CpG#22 | cg153684236 | .83±1.505 | 2.09±2.542 | .013 |
|  | CpG#23 | cg153684240 | 6.44±2.935 | 7.55±2.371 | .107 |
|  | CpG#24 | cg153684242 | 9.83±6.474 | 9.74±4.655 | .947 |
|  | CpG#25 | cg153684244 | 6.56±3.698 | 9.83±5.016 | .005 |
|  | CpG#26 | cg153684275 | 7.56±5.216 | 8.29±3.991 | .527 |
|  | CpG#27 | cg153684284 | 3.78±2.016 | 4.50±2.371 | .247 |
|  | CpG#28 | cg153688942 | 2.94±1.798 | 4.34±2.686 | .015 |
| *TLR6* gene body | CpG#1 | cg13006575 | 56.61±9.432 | 66.26±5.565 | <.001 |
|  | CpG#2 | cg13006591 | 91.61±6.572 | 91.21±4.254 | .760 |
|  | CpG#3 | cg25769980 | 87.28±6.952 | 90.28±2.996 | .091 |
| Protein expression | TLR2 |  | 178.4035±12.93122 | 703.8781±898.09597 | <.001 |
|  | TLR6 |  | 1.3219±.40024 | 12.9591±3.29140 | <.001 |
