# Supplementary material for: Aberrant DNA methylation of the toll-like receptors 2 and 6 genes in patients with obstructive sleep apnea
Source: PLoS One. 2020 Feb 18;15(2):e0228958. doi: 10.1371/journal.pone.0228958 (PMC7028278; doi:10.1371/journal.pone.0228958)
Supplement: S3 Table — (DOCX) [file pone.0228958.s008.docx]

**S3 Table. Multivariate linear regression with hierarchical comparisons showed that OSA is the independent risk factor of DNA methylation levels over CpG site #1, #2, #3, #18, #25 and #28 of the *TLR2* promoter region, CpG site #1 and #3 of *TLR6* gene body, and protein expression of TLR6.**

|  |  | Model 1 Demography | | | Model 2 OSA | | | Coefficients | | |
| --- | --- | --- | --- | --- | --- | --- | --- | --- | --- | --- |
|  |  | *F* | *p* | *R^2^* | *△F* | *p* | *△R^2^* | β | *t* | *pr^2^* |
| *TLR2* promoter region | CpG#1 | 1.664 | .089 | .291 | 4.546 | .037 | .051 | .327 | 2.132 | .071 |
|  | CpG#2 | 1.937 | .037 | .326 | 5.202 | .026 | .055 | .339 | 2.281 | .081 |
|  | CpG#3 | 2.179 | .017 | .353 | 5.293 | .025 | .053 | .335 | 2.301 | .082 |
|  | CpG#4 | 1.205 | .293 | .232 | .072 | .790 | .001 | -.004 | -.268 | .001 |
|  | CpG#5 | .953 | .514 | .192 | 2.421 | .125 | .032 | .259 | 1.556 | .040 |
|  | CpG#6 | 1.797 | .056 | .310 | 3.478 | .067 | .038 | .284 | 1.865 | .056 |
|  | CpG#7 | 1.912 | .040 | .323 | 2.668 | .108 | .029 | .248 | 1.633 | .043 |
|  | CpG#8 | 1.138 | .345 | ,221 | 1.521 | .222 | .020 | .203 | 1.233 | .025 |
|  | CpG#9 | .519 | .920 | .115 | 2.941 | .092 | .042 | .297 | 1.715 | .048 |
|  | CpG#10 | 1.097 | .378 | .215 | 2.300 | .135 | .029 | .249 | 1.516 | .038 |
|  | CpG#11 | 1.702 | .075 | .298 | 2.840 | .097 | .032 | .260 | 1.685 | .046 |
|  | CpG#12 | .662 | .811 | .142 | 2.594 | .113 | .036 | .276 | 1.611 | .042 |
|  | CpG#13 | 1.479 | .142 | .270 | 2.936 | .092 | .035 | .270 | 1.713 | .048 |
|  | CpG#14 | .982 | .484 | .197 | .392 | .534 | .005 | .106 | .626 | .007 |
|  | CpG#15 | 1.387 | .183 | .257 | 2.408 | .126 | .029 | .247 | 1.552 | .039 |
|  | CpG#16 | 1.135 | .347 | .221 | 1.852 | .179 | .024 | .223 | 1.361 | .030 |
|  | CpG#17 | .890 | .579 | .182 | 1.396 | .242 | .019 | .199 | 1.182 | .023 |
|  | CpG#18 | .929 | .538 | .188 | 4.333 | .042 | .056 | -.342 | -2.082 | .069 |
|  | CpG#19 | .838 | .633 | .173 | 1.577 | .214 | .022 | .213 | 1.256 | .026 |
|  | CpG#20 | 1.012 | .456 | .202 | .429 | .515 | .006 | -.110 | -.655 | .007 |
|  | CpG#21 | .436 | .961 | .098 | .270 | .605 | .004 | -.093 | -.519 | .004 |
|  | CpG#22 | 1.209 | .291 | .232 | 1.429 | .237 | .018 | .195 | 1.195 | .024 |
|  | CpG#23 | .902 | .565 | .184 | .310 | .580 | .004 | .095 | .556 | .005 |
|  | CpG#24 | .854 | .616 | .176 | .639 | .427 | .009 | .136 | .799 | .010 |
|  | CpG#25 | 1.301 | .230 | .245 | 6.221 | .015 | .072 | .389 | 2.494 | .095 |
|  | CpG#26 | .890 | .579 | .182 | .790 | .378 | .011 | .151 | .889 | .013 |
|  | CpG#27 | .514 | .923 | .114 | 3.000 | .089 | .043 | .300 | 1.732 | .048 |
|  | CpG#28 | 1.176 | .315 | .227 | 5.856 | .019 | .070 | .383 | 2.420 | .090 |
| *TLR6* gene body | CpG#1 | 1.419 | .168 | .262 | 20.736 | *<*.001 | .192 | .635 | 4.554 | .260 |
|  | CpG#2 | 1.239 | .270 | .236 | 1.057 | .308 | .013 | -.168 | -1.028 | .018 |
|  | CpG#3 | .594 | .868 | .129 | 9.421 | .003 | .120 | .502 | 3.069 | .138 |
| Protein expression | TLR2 | 1.002 | .467 | .215 | 2.407 | .127 | .034 | .265 | 1.551 | .043 |
|  | TLR6 | 3.661 | <.001 | .496 | 97.805 | *<*.001 | .325 | .825 | 9.890 | .645 |
