# Supplementary material for: Aberrant DNA methylation of the toll-like receptors 2 and 6 genes in patients with obstructive sleep apnea
Source: PLoS One. 2020 Feb 18;15(2):e0228958. doi: 10.1371/journal.pone.0228958 (PMC7028278; doi:10.1371/journal.pone.0228958)
Supplement: S4 Table — (DOCX) [file pone.0228958.s009.docx]

**S4 Table. Multivariate linear regression with hierarchical comparisons showed that AHI is the independent risk factor of DNA methylation levels over CpG site #1, #2, #3, #11, #12, #13, #15, #19, and #20 of the *TLR2* promoter, CpG site #1 of *TLR6* gene body, and protein expressions of TLR6**

|  |  | Model 1 Demography | | | Model 2 AHI | | | Coefficients | | |
| --- | --- | --- | --- | --- | --- | --- | --- | --- | --- | --- |
|  |  | *F* | *p* | *R^2^* | *△F* | *p* | *△R^2^* | β | *t* | *pr^2^* |
| *TLR2* promoter region | CpG#1 | 1.568 | .113 | .296 | 6.987 | .011 | .079 | .403 | 2.643 | .113 |
|  | CpG#2 | 1.568 | .049 | .332 | 4.632 | .036 | .052 | .326 | 2.152 | .078 |
|  | CpG#3 | 2.266 | .014 | .378 | 9.099 | .004 | .088 | .425 | 3.016 | .142 |
|  | CpG#4 | 1.205 | ,296 | .244 | .891 | .349 | .012 | -.157 | -.944 | .016 |
|  | CpG#5 | .931 | .536 | .200 | 2.293 | .136 | .032 | .256 | 1.514 | .040 |
|  | CpG#6 | 1.736 | .070 | .317 | 1.879 | .176 | .023 | .215 | 1.371 | .033 |
|  | CpG#7 | 1.774 | .062 | .322 | 1.487 | .228 | .018 | .191 | 1.220 | .026 |
|  | CpG#8 | 1.164 | .326 | .238 | 1.935 | .170 | .026 | .230 | 1.391 | .034 |
|  | CpG#9 | .492 | .935 | .116 | 1.548 | .219 | .024 | .223 | 1.244 | .027 |
|  | CpG#10 | 1.419 | .171 | .275 | 1.666 | .202 | .021 | .209 | 1.291 | .029 |
|  | CpG#11 | 1.785 | .060 | .323 | 6.191 | .016 | .068 | .374 | 2.488 | .101 |
|  | CpG#12 | .692 | .781 | .156 | 4.402 | .041 | .063 | .358 | 2.098 | .074 |
|  | CpG#13 | 1.374 | .193 | .269 | 7.455 | .008 | .087 | .423 | 2.730 | .120 |
|  | CpG#14 | .938 | .529 | .201 | .625 | .433 | .009 | .136 | .791 | .011 |
|  | CpG#15 | 1.334 | .214 | .263 | 5.926 | .018 | .072 | .383 | 2.434 | .097 |
|  | CpG#16 | .995 | .473 | .210 | 2.188 | .145 | .030 | .249 | 1.479 | .084 |
|  | CpG#17 | .967 | .500 | .206 | 1.474 | .230 | .021 | .206 | 1.214 | .026 |
|  | CpG#18 | .793 | .680 | .175 | 2.874 | .096 | .041 | -.290 | -1.695 | .050 |
|  | CpG#19 | .795 | .678 | .175 | 4.210 | .045 | .059 | .347 | 2.052 | .071 |
|  | CpG#20 | .896 | .573 | .194 | 6.367 | .015 | .084 | -.414 | -2.523 | .104 |
|  | CpG#21 | .380 | .979 | .092 | 3.078 | .085 | .048 | -.314 | -1.755 | .053 |
|  | CpG#22 | 1.234 | .2775 | .248 | 2.423 | .125 | .032 | .255 | 1.557 | .042 |
|  | CpG#23 | .839 | .632 | .184 | .214 | .645 | .003 | -.081 | -.463 | .004 |
|  | CpG#24 | .963 | .505 | .205 | .548 | .462 | .008 | .127 | .741 | .010 |
|  | CpG#25 | 1.213 | .290 | .245 | 1.226 | .273 | .016 | .184 | 1.107 | .022 |
|  | CpG#26 | .774 | .699 | .172 | 2.095 | .153 | .030 | .250 | 1.447 | .037 |
|  | CpG#27 | .406 | .971 | .098 | .618 | .435 | .010 | .143 | .786 | .011 |
|  | CpG#28 | 1.134 | .349 | .233 | 1.354 | .250 | .018 | .194 | 1.164 | .024 |
| *TLR6* gene body | CpG#1 | 1.336 | .213 | .264 | 19.875 | *<*.001 | .195 | .633 | 4.458 | .265 |
|  | CpG#2 | 1.414 | .173 | .275 | .313 | *.578* | .004 | -.092 | -.559 | .006 |
|  | CpG#3 | .782 | .252 | .173 | 1.343 | *.252* | .020 | .201 | 1.159 | .024 |
| Protein expression | TLR2 | .912 | .557 | .211 | .774 | .383 | .012 | .161 | .880 | .015 |
|  | TLR6 | 3.066 | .001 | .474 | 37.501 | *<*.001 | .225 | .698 | 6.124 | .429 |
