# Supplementary material for: Aberrant DNA methylation of the toll-like receptors 2 and 6 genes in patients with obstructive sleep apnea
Source: PLoS One. 2020 Feb 18;15(2):e0228958. doi: 10.1371/journal.pone.0228958 (PMC7028278; doi:10.1371/journal.pone.0228958)
Supplement: S5 Table — (DOCX) [file pone.0228958.s010.docx]

**S5 Table. Multivariate linear regression with hierarchical comparisons showed that ODI is the independent risk factor of DNA methylation levels over CpG site #1, #2, #3, #8, #11, #12, #13, #15, #17, #19, and #22 of the *TLR2* promoter, CpG site #1 of *TLR6* gene body, and protein expressions of TLR6**

|  |  | Model 1 Demography | | | Model 2 ODI | | | Coefficients | | |
| --- | --- | --- | --- | --- | --- | --- | --- | --- | --- | --- |
|  |  | *F* | *p* | *R^2^* | *△F* | *p* | *△R^2^* | β | *t* | *pr^2^* |
| *TLR2* promoter region | CpG#1 | 1.700 | .075 | .295 | 10.745 | .002 | .107 | .462 | 3.278 | .152 |
|  | CpG#2 | 2.081 | .023 | .338 | 8.379 | .004 | .084 | .409 | 2.956 | .127 |
|  | CpG#3 | 2.150 | .019 | .346 | 14.667 | *<*.001 | .129 | .506 | 3.831 | .196 |
|  | CpG#4 | 1.196 | .299 | .227 | .165 | .686 | .002 | -.065 | -.406 | .003 |
|  | CpG#5 | 1.004 | .463 | .198 | 3.666 | .060 | .046 | .303 | 1.915 | .058 |
|  | CpG#6 | 1.819 | .052 | .309 | 3.371 | .071 | .037 | .270 | 1.836 | .053 |
|  | CpG#7 | 1.946 | .035 | .324 | 3.690 | .060 | .039 | .279 | 1.921 | .058 |
|  | CpG#8 | 1.188 | .036 | .226 | 10.872 | .002 | .119 | .486 | 3.297 | .154 |
|  | CpG#9 | .528 | .915 | .115 | 3.630 | .062 | .050 | .317 | 1.905 | .057 |
|  | CpG#10 | 1.293 | .235 | .241 | 2.809 | .099 | .034 | .260 | 1.676 | .045 |
|  | CpG#11 | 1.701 | .075 | .295 | 12.321 | .001 | .120 | .489 | 3.510 | .171 |
|  | CpG#12 | .620 | .847 | .132 | 12.379 | .001 | .148 | .543 | 3.518 | .171 |
|  | CpG#13 | 1.470 | .145 | .266 | 10.717 | .002 | .111 | .471 | 3.274 | .151 |
|  | CpG#14 | .990 | .476 | .196 | 1.343 | .251 | .018 | .187 | 1.159 | .022 |
|  | CpG#15 | 1.236 | .271 | .233 | 14.177 | <.001 | .147 | .540 | 3.765 | .191 |
|  | CpG#16 | .880 | ,589 | .178 | 2.863 | .096 | .037 | .273 | 1.692 | .045 |
|  | CpG#17 | .860 | .610 | .175 | 4.146 | .046 | .053 | .326 | 2.036 | .065 |
|  | CpG#18 | .821 | .651 | .168 | .950 | .334 | .013 | -.161 | -.975 | .016 |
|  | CpG#19 | .875 | .595 | .177 | 6.817 | .011 | .084 | .409 | 2.611 | .102 |
|  | CpG#20 | 1.063 | .408 | .207 | 1.406 | .240 | .018 | -.190 | -1.186 | .023 |
|  | CpG#21 | .392 | .976 | .088 | 2.171 | .146 | .032 | -.252 | -1.473 | .035 |
|  | CpG#22 | 1.241 | .268 | .234 | 4.687 | .034 | .056 | .332 | 2.165 | .072 |
|  | CpG#23 | .963 | .503 | .192 | .083 | .774 | .001 | .047 | .288 | .001 |
|  | CpG#24 | .837 | .634 | .171 | .092 | .762 | .001 | .050 | .304 | .002 |
|  | CpG#25 | 1.358 | .197 | .250 | .959 | .331 | .012 | .153 | .979 | .016 |
|  | CpG#26 | .888 | .581 | .179 | .011 | .917 | <.001 | .017 | .104 | <.001 |
|  | CpG#27 | .538 | .909 | .117 | .618 | .435 | .009 | .134 | .786 | .010 |
|  | CpG#28 | 1.201 | .296 | .228 | 2.710 | .105 | .033 | .258 | 1.646 | .043 |
| *TLR6* gene body | CpG#1 | 1.380 | .187 | .253 | 6.994 | .010 | .078 | .394 | 2.645 | .104 |
|  | CpG#2 | 1.007 | .460 | .199 | .033 | .856 | <.001 | .030 | .182 | <.001 |
|  | CpG#3 | .576 | .882 | .124 | 2.130 | .150 | .030 | .244 | 1.459 | .034 |
| Protein expression | TLR2 | 1.022 | .448 | .215 | .926 | .340 | .013 | .165 | .962 | .017 |
|  | TLR6 | 3.722 | <.001 | .499 | 20.954 | *<*.001 | .138 | .538 | 4.578 | .276 |
