# Supplementary material for: Aberrant DNA methylation of the toll-like receptors 2 and 6 genes in patients with obstructive sleep apnea
Source: PLoS One. 2020 Feb 18;15(2):e0228958. doi: 10.1371/journal.pone.0228958 (PMC7028278; doi:10.1371/journal.pone.0228958)
Supplement: S6 Table — A q value threshold of 0.1 was selected to separate false from true discoveries, and the first 2 would be significant. (DOCX) [file pone.0228958.s011.docx]

**S6 Table. Multiple comparisons of DNA methylation levels in OSA. A *q* value threshold of 0.1 was selected to separate false from true discoveries, and the first 2 would be significant.**

|  | *p* | *Rank* | *q* |
| --- | --- | --- | --- |
| *TLR6* CpG#1 | 0.000027 | 1 | 0.0004455 |
| *TLR6* CpG#3 | 0.003000 | 2 | 0.0330000 |
| *TLR2* CpG#25 | 0.015000 | 3 | 0.1225714 |
| *TLR2* CpG#28 | 0.019000 | 4 | 0.1225714 |
| *TLR2* CpG#3 | 0.025000 | 5 | 0.1225714 |
| *TLR2* CpG#2 | 0.026000 | 6 | 0.1225714 |
| *TLR2* CpG#1 | 0.037000 | 7 | 0.1526250 |
| *TLR2* CpG#18 | 0.042000 | 8 | 0.1540000 |
| *TLR2* CpG#6 | 0.067000 | 9 | 0.2205789 |
| *TLR2* CpG#27 | 0.089000 | 10 | 0.2205789 |
| *TLR2* CpG#9 | 0.092000 | 11 | 0.2205789 |
| *TLR2* CpG#13 | 0.092000 | 12 | 0.2205789 |
| *TLR2* CpG#11 | 0.097000 | 13 | 0.2205789 |
| *TLR2* CpG#7 | 0.108000 | 14 | 0.2205789 |
| *TLR2* CpG#12 | 0.113000 | 15 | 0.2205789 |
| *TLR2* CpG#5 | 0.125000 | 16 | 0.2205789 |
| *TLR2* CpG#15 | 0.126000 | 17 | 0.2205789 |
| *TLR2* CpG#10 | 0.135000 | 18 | 0.2227500 |
| *TLR2* CpG#16 | 0.179000 | 19 | 0.2812857 |
| *TLR2* CpG#19 | 0.214000 | 20 | 0.3185217 |
| *TLR2* CpG#8 | 0.222000 | 21 | 0.3185217 |
| *TLR2* CpG#22 | 0.237000 | 22 | 0.3194400 |
| *TLR2* CpG#17 | 0.242000 | 23 | 0.3194400 |
| *TLR6* CpG#2 | 0.308000 | 24 | 0.3909231 |
| *TLR2* CpG#26 | 0.378000 | 25 | 0.4620000 |
| *TLR2* CpG#24 | 0.427000 | 26 | 0.5032500 |
| *TLR2* CpG#20 | 0.515000 | 27 | 0.5860345 |
| *TLR2* CpG#14 | 0.534000 | 28 | 0.5874000 |
| *TLR2* CpG#23 | 0.580000 | 29 | 0.6174194 |
| *TLR2* CpG#21 | 0.605000 | 30 | 0.6239062 |
| *TLR2* CpG#4 | 0.790000 | 31 | 0.7900000 |
