# Supplementary material for: Aberrant DNA methylation of the toll-like receptors 2 and 6 genes in patients with obstructive sleep apnea
Source: PLoS One. 2020 Feb 18;15(2):e0228958. doi: 10.1371/journal.pone.0228958 (PMC7028278; doi:10.1371/journal.pone.0228958)
Supplement: S7 Table — A q value threshold of 0.1 was selected to separate false from true discoveries, and the first 7 would be significant. (DOCX) [file pone.0228958.s012.docx]

**S7 Table. Multiple comparisons of DNA methylation levels in AHI. A *q* value threshold of 0.1 was selected to separate false from true discoveries, and the first 7 would be significant.**

|  | *p* | *Rank* | *q* |
| --- | --- | --- | --- |
| *TLR6* CpG#1 | 0.000041 | 1 | 0.0006765 |
| *TLR2* CpG#3 | 0.004000 | 2 | 0.0440000 |
| *TLR2* CpG#13 | 0.008000 | 3 | 0.0660000 |
| *TLR2* CpG#1 | 0.011000 | 4 | 0.0726000 |
| *TLR2* CpG#20 | 0.015000 | 5 | 0.0742500 |
| *TLR2* CpG#11 | 0.016000 | 6 | 0.0742500 |
| *TLR2* CpG#15 | 0.018000 | 7 | 0.0742500 |
| *TLR2* CpG#2 | 0.036000 | 8 | 0.1320000 |
| *TLR2* CpG#12 | 0.041000 | 9 | 0.1350000 |
| *TLR2* CpG#19 | 0.045000 | 10 | 0.1350000 |
| *TLR2* CpG#21 | 0.085000 | 11 | 0.2337500 |
| *TLR2* CpG#18 | 0.096000 | 12 | 0.2436923 |
| *TLR2* CpG#22 | 0.125000 | 13 | 0.2946429 |
| *TLR2* CpG#5 | 0.136000 | 14 | 0.2970000 |
| *TLR2* CpG#16 | 0.145000 | 15 | 0.2970000 |
| *TLR2* CpG#26 | 0.153000 | 16 | 0.2970000 |
| *TLR2* CpG#8 | 0.170000 | 17 | 0.3056842 |
| *TLR2* CpG#6 | 0.176000 | 18 | 0.3056842 |
| *TLR2* CpG#10 | 0.202000 | 19 | 0.3300000 |
| *TLR2* CpG#9 | 0.219000 | 20 | 0.3300000 |
| *TLR2* CpG#7 | 0.228000 | 21 | 0.3300000 |
| *TLR2* CpG#17 | 0.230000 | 22 | 0.3300000 |
| *TLR2* CpG#28 | 0.250000 | 23 | 0.3326400 |
| *TLR6* CpG#3 | 0.252000 | 24 | 0.3326400 |
| *TLR2* CpG#25 | 0.273000 | 25 | 0.3465000 |
| *TLR2* CpG#4 | 0.349000 | 26 | 0.4265556 |
| *TLR2* CpG#14 | 0.433000 | 27 | 0.4785000 |
| *TLR2* CpG#27 | 0.435000 | 28 | 0.4785000 |
| *TLR2* CpG#24 | 0.462000 | 29 | 0.4918065 |
| *TLR6* CpG#2 | 0.578000 | 30 | 0.5960625 |
| *TLR2* CpG#23 | 0.645000 | 31 | 0.6450000 |
