# Supplementary material for: Aberrant DNA methylation of the toll-like receptors 2 and 6 genes in patients with obstructive sleep apnea
Source: PLoS One. 2020 Feb 18;15(2):e0228958. doi: 10.1371/journal.pone.0228958 (PMC7028278; doi:10.1371/journal.pone.0228958)
Supplement: S8 Table — A q value threshold of 0.1 was selected to separate false from true discoveries, and the first 11 would be significant. (DOCX) [file pone.0228958.s013.docx]

**S8 Table. Multiple comparisons of DNA methylation levels in ODI. A *q* value threshold of 0.1 was selected to separate false from true discoveries, and the first 11 would be significant.**

|  | *p* | *Rank* | *q* |
| --- | --- | --- | --- |
| *TLR2* CpG#3 | 0.000308 | 1 | 0.00419100 |
| *TLR2* CpG#15 | 0.000381 | 2 | 0.00419100 |
| *TLR2* CpG#11 | 0.001000 | 3 | 0.00660000 |
| *TLR2* CpG#12 | 0.001000 | 4 | 0.00660000 |
| *TLR2* CpG#1 | 0.002000 | 5 | 0.00825000 |
| *TLR2* CpG#8 | 0.002000 | 6 | 0.00825000 |
| *TLR2* CpG#13 | 0.002000 | 7 | 0.00825000 |
| *TLR2* CpG#2 | 0.004000 | 8 | 0.01466667 |
| *TLR6* CpG#1 | 0.010000 | 9 | 0.03300000 |
| *TLR2* CpG#19 | 0.011000 | 10 | 0.03300000 |
| *TLR2* CpG#22 | 0.034000 | 11 | 0.09350000 |
| *TLR2* CpG#17 | 0.046000 | 12 | 0.11676923 |
| *TLR2* CpG#5 | 0.060000 | 13 | 0.12787500 |
| *TLR2* CpG#7 | 0.060000 | 14 | 0.12787500 |
| *TLR2* CpG#9 | 0.062000 | 15 | 0.12787500 |
| *TLR2* CpG#6 | 0.071000 | 16 | 0.13782353 |
| *TLR2* CpG#16 | 0.096000 | 17 | 0.17194737 |
| *TLR2* CpG#10 | 0.099000 | 18 | 0.17194737 |
| *TLR2* CpG#28 | 0.105000 | 19 | 0.17325000 |
| *TLR2* CpG#21 | 0.146000 | 20 | 0.22500000 |
| *TLR6* CpG#3 | 0.150000 | 21 | 0.22500000 |
| *TLR2* CpG#20 | 0.240000 | 22 | 0.34434783 |
| *TLR2* CpG#14 | 0.251000 | 23 | 0.34512500 |
| *TLR2* CpG#25 | 0.331000 | 24 | 0.41555556 |
| *TLR2* CpG#18 | 0.334000 | 25 | 0.41555556 |
| *TLR2* CpG#27 | 0.435000 | 26 | 0.51267857 |
| *TLR2* CpG#4 | 0.686000 | 27 | 0.78062069 |
| *TLR2* CpG#24 | 0.762000 | 28 | 0.82393548 |
| *TLR2* CpG#23 | 0.774000 | 29 | 0.82393548 |
| *TLR6* CpG#2 | 0.856000 | 30 | 0.88275000 |
| *TLR2* CpG#26 | 0.917000 | 31 | 0.91700000 |
