# Supplementary material for: Aberrant DNA methylation of the toll-like receptors 2 and 6 genes in patients with obstructive sleep apnea
Source: PLoS One. 2020 Feb 18;15(2):e0228958. doi: 10.1371/journal.pone.0228958 (PMC7028278; doi:10.1371/journal.pone.0228958)
Supplement: S9 Table — (DOCX) [file pone.0228958.s014.docx]

S9 Table. DNA methylation levels of *TLR2* promoter region, *TLR6* gene body, and protein expressions of TLR2 and TLR6 in OSA without and with EDS. **(**genome build “GRCh38.p13”)

|  |  |  | Non-EDS (n=18) | EDS (n=40) | *p* |
| --- | --- | --- | --- | --- | --- |
| *TLR2* promoter region | CpG#1 | cg153684036 | 16.95±3.501 | 15.94±7.824 | .722 |
|  | CpG#2 | cg153684048 | 19.17±10.867 | 16.36±8.871 | .302 |
|  | CpG#3 | cg153684062 | 16.44±6.604 | 4.61±5.430 | .272 |
|  | CpG#4 | cg153684076 | 7.33±3.361 | 8.23±2.939 | .311 |
|  | CpG#5 | cg153684086 | 5.78±4.346 | 5.70±3.458 | .942 |
|  | CpG#6 | cg153684106 | 5.98±2.272 | 5.73±2.653 | .733 |
|  | CpG#7 | cg153684110 | 5.89±3.998 | 4.80±2.544 | .215 |
|  | CpG#8 | cg153684112 | 3.33±1.645 | 3.83±2.159 | .394 |
|  | CpG#9 | cg153684120 | 10.40±14.942 | 12.00±20.820 | .771 |
|  | CpG#10 | cg153684124 | 2.46±.938 | 2.28±.614 | .472 |
|  | CpG#11 | cg153684148 | 3.33±1.283 | 3.38±1.596 | .923 |
|  | CpG#12 | cg153684150 | 4.01±2.045 | 3.68±1.446 | .472 |
|  | CpG#13 | cg153684169 | 4.33±2.952 | 3.37±1.897 | .220 |
|  | CpG#14 | cg153684175 | 3.67±1.414 | 4.33±2.076 | .225 |
|  | CpG#15 | cg153684180 | 4.20±2.209 | 4.57±1.868 | .507 |
|  | CpG#16 | cg153684183 | 6.13±2.225 | 5.84±1.679 | .581 |
|  | CpG#17 | cg153684187 | 4.33±1.886 | 4.14±1.684 | .706 |
|  | CpG#18 | cg153684194 | 5.93±2.549 | 6.94±2.433 | .154 |
|  | CpG#19 | cg153684205 | 3.51±3.538 | 2.42±2.104 | .150 |
|  | CpG#20 | cg153684212 | 6.15±2.336 | 6.77±2.369 | .357 |
|  | CpG#21 | cg153684232 | 3.88±1.121 | 4.15±1.548 | .513 |
|  | CpG#22 | cg153684236 | 2.20±2.681 | 2.05±2.511 | .837 |
|  | CpG#23 | cg153684240 | 6.83±1.295 | 7.88±2.672 | .051 |
|  | CpG#24 | cg153684242 | 10.56±4.076 | 9.38±4.897 | .376 |
|  | CpG#25 | cg153684244 | 10.17±3.092 | 9.68±5.704 | .673 |
|  | CpG#26 | cg153684275 | 9.39±4.408 | 7.80±3.743 | .163 |
|  | CpG#27 | cg153684284 | 5.11±2.111 | 4.23±2.455 | .190 |
|  | CpG#28 | cg153688942 | 4.28±2.270 | 4.38±2.880 | .900 |
| *TLR6* gene body | CpG#1 | cg13006575 | 67.50±5.803 | 65.70±5.436 | .258 |
|  | CpG#2 | cg13006591 | 88.89±5.890 | 92.25±2.780 | .032 |
|  | CpG#3 | cg25769980 | 89.56±2.684 | 90.60±3.103 | .222 |
| Protein expression | TLR2 |  | 771.66±3.103 | 677.12±3.103 | .734 |
|  | TLR6 |  | 12.58±3.103 | 13.10±3.103 | .606 |
