# Supplementary material for: Aberrant DNA methylation of the toll-like receptors 2 and 6 genes in patients with obstructive sleep apnea
Source: PLoS One. 2020 Feb 18;15(2):e0228958. doi: 10.1371/journal.pone.0228958 (PMC7028278; doi:10.1371/journal.pone.0228958)
Supplement: S10 Table — (DOCX) [file pone.0228958.s015.docx]

**S10 Table. Multivariate linear regression with hierarchical comparisons showed that EDS is the independent risk factor of DNA methylation levels over CpG site #2 of *TLR6* gene body.**

|  |  | Model 1 Demography | | | Model 2 EDS | | | Coefficients | | |
| --- | --- | --- | --- | --- | --- | --- | --- | --- | --- | --- |
|  |  | *F* | *p* | *R^2^* | *△F* | *p* | *△R^2^* | *β* | *t* | *pr^2^* |
| *TLR2* promoter region | CpG#1 | 1.461 | .165 | .343 | <.001 | .996 | <.001 | -.001 | -.005 | <.001 |
|  | CpG#2 | 1.563 | .127 | .358 | .049 | .825 | .001 | -.031 | -.222 | .001 |
|  | CpG#3 | 1.901 | .051 | .404 | .406 | .527 | .006 | -.086 | -.637 | .001 |
|  | CpG#4 | .906 | .564 | .244 | 1.408 | .242 | .025 | .179 | 1.187 | .033 |
|  | CpG#5 | .842 | .628 | .231 | .002 | .969 | <.001 | -.006 | -.040 | <.001 |
|  | CpG#6 | 1.738 | .080 | .383 | .317 | .576 | .005 | .078 | .563 | .008 |
|  | CpG#7 | 1.954 | .045 | .411 | <.001 | .986 | <.001 | -.002 | -.018 | <.001 |
|  | CpG#8 | .996 | .477 | .262 | 3.521 | .068 | .058 | .272 | 1.876 | .079 |
|  | CpG#9 | .514 | .919 | .155 | .792 | .379 | .016 | .143 | .890 | .019 |
|  | CpG#10 | 1.321 | .233 | .321 | .487 | .489 | .008 | -.101 | -.698 | .012 |
|  | CpG#11 | 1.368 | .208 | .328 | .023 | .881 | <.001 | .022 | .150 | .001 |
|  | CpG#12 | .478 | .939 | .146 | .309 | .581 | .006 | -.090 | -.556 | .007 |
|  | CpG#13 | 1.387 | .198 | .331 | 1.551 | .220 | .024 | -.176 | -1.245 | .036 |
|  | CpG#14 | .775 | .696 | .217 | .347 | .559 | .007 | .091 | .589 | .008 |
|  | CpG#15 | 1.122 | .368 | .286 | .241 | .626 | .004 | .073 | .491 | .006 |
|  | CpG#16 | 1.212 | ,301 | .302 | .016 | .899 | <.001 | -.019 | -.128 | <.001 |
|  | CpG#17 | .958 | .513 | .255 | .032 | .859 | .001 | -.027 | -.179 | .001 |
|  | CpG#18 | 1.297 | .247 | .317 | 3.219 | .080 | .050 | .252 | 1.794 | .073 |
|  | CpG#19 | .611 | .849 | .179 | 1.910 | .174 | .037 | -.216 | -1.382 | .045 |
|  | CpG#20 | .825 | .646 | .228 | .187 | .667 | .004 | .067 | .433 | .004 |
|  | CpG#21 | .577 | .876 | .171 | .471 | .497 | .009 | .109 | .686 | .011 |
|  | CpG#22 | 1.042 | .435 | .271 | <.001 | .988 | <.001 | -.002 | -.016 | <.001 |
|  | CpG#23 | .642 | .823 | .187 | .959 | .333 | .019 | .154 | .979 | .023 |
|  | CpG#24 | 1.135 | .358 | .288 | .893 | .350 | .015 | -.139 | -.945 | .021 |
|  | CpG#25 | 1.454 | .168 | .342 | .002 | .967 | <.001 | .006 | .042 | <.001 |
|  | CpG#26 | 1.161 | .337 | .293 | 1.460 | .234 | .024 | -.176 | -1.208 | .034 |
|  | CpG#27 | .793 | .678 | .221 | 1.590 | .214 | .029 | -.192 | -1.261 | .037 |
|  | CpG#28 | 1.277 | .259 | .313 | .201 | .656 | .003 | .065 | .449 | .005 |
| *TLR6* gene body | CpG#1 | 1.246 | .278 | .308 | .023 | .880 | <.001 | -.022 | -.153 | .001 |
|  | CpG#2 | 2.046 | .035 | .422 | 7.469 | .009 | .089 | .337 | 2.733 | .153 |
|  | CpG#3 | .806 | .665 | .224 | .310 | .580 | .006 | .086 | .557 | .008 |
| Protein expression | TLR2 | .594 | .860 | .194 | .114 | .738 | .003 | .059 | .337 | .003 |
|  | TLR6 | .919 | .551 | .272 | .100 | .754 | .002 | .053 | .316 | .003 |
