# Supplementary material for: Aberrant DNA methylation of the toll-like receptors 2 and 6 genes in patients with obstructive sleep apnea
Source: PLoS One. 2020 Feb 18;15(2):e0228958. doi: 10.1371/journal.pone.0228958 (PMC7028278; doi:10.1371/journal.pone.0228958)
Supplement: S11 Table — A q value threshold of 0.1 was selected to separate false from true discoveries. (DOCX) [file pone.0228958.s016.docx]

**S11 Table. Multiple comparisons of DNA methylation levels in EDS. A *q* value threshold of 0.1 was selected to separate false from true discoveries.**

|  | *p* | *Rank* | *q* |
| --- | --- | --- | --- |
| *TLR6* CpG#2 | 0.009 | 1 | 0.297 |
| *TLR2* CpG#8 | 0.068 | 2 | 0.880 |
| *TLR2* CpG#18 | 0.080 | 3 | 0.880 |
| *TLR2* CpG#19 | 0.174 | 4 | 0.996 |
| *TLR2* CpG#27 | 0.214 | 5 | 0.996 |
| *TLR2* CpG#13 | 0.220 | 6 | 0.996 |
| *TLR2* CpG#26 | 0.234 | 7 | 0.996 |
| *TLR2* CpG#4 | 0.242 | 8 | 0.996 |
| *TLR2* CpG#23 | 0.333 | 9 | 0.996 |
| *TLR2* CpG#24 | 0.350 | 10 | 0.996 |
| *TLR2* CpG#9 | 0.379 | 11 | 0.996 |
| *TLR2* CpG#10 | 0.489 | 12 | 0.996 |
| *TLR2* CpG#21 | 0.497 | 13 | 0.996 |
| *TLR2* CpG#3 | 0.527 | 14 | 0.996 |
| *TLR2* CpG#13 | 0.559 | 15 | 0.996 |
| *TLR2* CpG#6 | 0.576 | 16 | 0.996 |
| *TLR6* CpG#3 | 0.580 | 17 | 0.996 |
| *TLR2* CpG#12 | 0.581 | 18 | 0.996 |
| *TLR2* CpG#15 | 0.626 | 19 | 0.996 |
| *TLR2* CpG#28 | 0.656 | 20 | 0.996 |
| *TLR2* CpG#20 | 0.667 | 21 | 0.996 |
| *TLR2* CpG#2 | 0.825 | 22 | 0.996 |
| *TLR2* CpG#17 | 0.859 | 23 | 0.996 |
| *TLR6* CpG#1 | 0.880 | 24 | 0.996 |
| *TLR2* CpG#11 | 0.881 | 25 | 0.996 |
| *TLR2* CpG#16 | 0.899 | 26 | 0.996 |
| *TLR2* CpG#25 | 0.967 | 27 | 0.996 |
| *TLR2* CpG#5 | 0.969 | 28 | 0.996 |
| *TLR2* CpG#7 | 0.986 | 29 | 0.996 |
| *TLR2* CpG#22 | 0.988 | 30 | 0.996 |
| *TLR2* CpG#1 | 0.996 | 31 | 0.996 |
