# Supplementary material for: Aberrant DNA methylation of the toll-like receptors 2 and 6 genes in patients with obstructive sleep apnea
Source: PLoS One. 2020 Feb 18;15(2):e0228958. doi: 10.1371/journal.pone.0228958 (PMC7028278; doi:10.1371/journal.pone.0228958)
Supplement: S12 Table — (DOCX) [file pone.0228958.s017.docx]

**S12 Table. Difference of DNA methylation levels in OSA before and after CPAP management. (**genome build “GRCh38.p13”)

|  |  |  | Before | After | Difference | *p* |
| --- | --- | --- | --- | --- | --- | --- |
| *TLR2* promoter region | CpG#1 | cg153684036 | 12.00±2.70 | 8.142±.89 | 3.85±3.18 | .018 |
|  | CpG#2 | cg153684048 | 11.86±2.19 | 11.42±2.22 | .42±3.77 | .774 |
|  | CpG#3 | cg153684062 | 12.00±3.10 | 10.00±1.15 | 2.00±3.00 | .128 |
|  | CpG#4 | cg153684076 | 7.86±2.19 | 7.71±2.49 | .14±3.80 | .924 |
|  | CpG#5 | cg153684086 | 3.71±1.11 | 4.42±1.61 | -.714±1.88 | .356 |
|  | CpG#6 | cg153684106 | 4.00±1.63 | 5.14±.89 | -1.14±1.67 | .121 |
|  | CpG#7 | cg153684110 | 3.29±.75 | 4.00±.81 | -.71±1.38 | .220 |
|  | CpG#8 | cg153684112 | 3.14±.90 | 2.14±1.21 | 1.00±1.63 | .156 |
|  | CpG#9 | cg153684120 | 4.86±1.06 | 2.42±1.27 | 2.42±1.90 | .015 |
|  | CpG#10 | cg153684124 | 1.86±.69 | 1.85±.69 | .00±.81 | 1.000 |
|  | CpG#11 | cg153684148 | 2.57±1.39 | 2.42±.78 | .142±1.34 | .788 |
|  | CpG#12 | cg153684150 | 3.14±1.06 | 2.42±1.27 | .71±1.97 | .376 |
|  | CpG#13 | cg153684169 | 2.71±.75 | 3.00±1.41 | -.28±1.49 | .631 |
|  | CpG#14 | cg153684175 | 3.71±.95 | 3.00±1.29 | .71±1.70 | .310 |
|  | CpG#15 | cg153684180 | 3.86±1.34 | 3.42±1.27 | .42±1.71 | .534 |
|  | CpG#16 | cg153684183 | 6.00±1.15 | 5.00±.81 | 1.00±1.41 | .111 |
|  | CpG#17 | cg153684187 | 4.14±1.77 | 3.28±1.38 | .85±2.03 | .308 |
|  | CpG#18 | cg153684194 | 8.00±1.41 | 7.00±1.52 | 1.00±2.30 | .296 |
|  | CpG#19 | cg153684205 | 1.57±.78 | 1.85±.37 | -.285±.95 | .457 |
|  | CpG#20 | cg153684212 | 6.00±1.52 | 4.85±1.46 | 1.14±2.26 | .231 |
|  | CpG#21 | cg153684232 | 3.71±.75 | 3.28±.48 | .42±.78 | .200 |
|  | CpG#22 | cg153684236 | 1.57±2.07 | .28±.75 | 1.28±1.60 | .078 |
|  | CpG#23 | cg153684240 | 7.71±2.28 | 4.42±.53 | 3.28±2.05 | .006 |
|  | CpG#24 | cg153684242 | 7.14±2.91 | 15.00±7.30 | -7.85±7.22 | .028 |
|  | CpG#25 | cg153684244 | 6.43±4.07 | 7.28±4.15 | -.854±5.89 | 714 |
|  | CpG#26 | cg153684275 | 5.57±2.07 | 12.00±5.83 | -6.42±6.60 | .042 |
|  | CpG#27 | cg153684284 | 3.00±1.73 | 5.00±2.30 | -2.00±3.10 | .140 |
|  | CpG#28 | cg153688942 | 2.43±1.81 | 3.4286±1.61 | -1.00±2.76 | .376 |
| *TLR6* gene body | CpG#1 | cg13006575 | 70.14±3.89 | 53.86±4.38 | 16.29±3.45 | <.001 |
|  | CpG#2 | cg13006591 | 88.71±4.89 | 85.86±7.63 | 2.86±7.78 | .369 |
|  | CpG#3 | cg25769980 | 90.29±1.38 | 85.00±2.71 | 5.29±2.93 | .003 |
| Protein expression | TLR2 |  | 706.53±73.29 | 268.04±26.62 | 438.49±69.84 | <.001 |
|  | TLR6 |  | 11.59±2.57 | 3.70±.86 | 7.89±1.84 | <.001 |
