# Supplementary material for: Aberrant DNA methylation of the toll-like receptors 2 and 6 genes in patients with obstructive sleep apnea
Source: PLoS One. 2020 Feb 18;15(2):e0228958. doi: 10.1371/journal.pone.0228958 (PMC7028278; doi:10.1371/journal.pone.0228958)
Supplement: S13 Table — A q value threshold of 0.1 was selected to separate false from true discoveries, and the first 5 would be significant. (DOCX) [file pone.0228958.s018.docx]

**S13 Table. Multiple comparisons of DNA methylation levels in CPAP management. A *q* value threshold of 0.1 was selected to separate false from true discoveries, and the first 5 would be significant.**

|  | *p* | *Rank* | *q* |
| --- | --- | --- | --- |
| *TLR6* CpG#1 | 0.000016 | 1 | 0.00026400 |
| *TLR6* CpG#3 | 0.003000 | 2 | 0.02475000 |
| *TLR2* CpG#23 | 0.006000 | 3 | 0.03960000 |
| *TLR2* CpG#9 | 0.015000 | 4 | 0.08250000 |
| *TLR2* CpG#1 | 0.018000 | 5 | 0.08485714 |
| *TLR2* CpG#24 | 0.028000 | 6 | 0.11550000 |
| *TLR2* CpG#26 | 0.042000 | 7 | 0.15400000 |
| *TLR2* CpG#22 | 0.078000 | 8 | 0.25740000 |
| *TLR2* CpG#16 | 0.111000 | 9 | 0.32492308 |
| *TLR2* CpG#6 | 0.121000 | 10 | 0.32492308 |
| *TLR2* CpG#3 | 0.128000 | 11 | 0.32492308 |
| *TLR2* CpG#27 | 0.140000 | 12 | 0.33000000 |
| *TLR2* CpG#8 | 0.156000 | 13 | 0.34320000 |
| *TLR2* CpG#21 | 0.200000 | 14 | 0.41250000 |
| *TLR2* CpG#7 | 0.220000 | 15 | 0.42350000 |
| *TLR2* CpG#20 | 0.231000 | 16 | 0.42350000 |
| *TLR2* CpG#18 | 0.296000 | 17 | 0.48714286 |
| *TLR2* CpG#17 | 0.308000 | 18 | 0.48714286 |
| *TLR2* CpG#14 | 0.310000 | 19 | 0.48714286 |
| *TLR2* CpG#5 | 0.356000 | 20 | 0.49632000 |
| *TLR6* CpG#2 | 0.369000 | 21 | 0.49632000 |
| *TLR2* CpG#12 | 0.376000 | 22 | 0.49632000 |
| *TLR2* CpG#28 | 0.376000 | 23 | 0.49632000 |
| *TLR2* CpG#19 | 0.457000 | 24 | 0.58003846 |
| *TLR2* CpG#15 | 0.534000 | 25 | 0.65266667 |
| *TLR2* CpG#13 | 0.631000 | 26 | 0.74367857 |
| *TLR2* CpG#25 | 0.714000 | 27 | 0.81248276 |
| *TLR2* CpG#2 | 0.774000 | 28 | 0.83883871 |
| *TLR2* CpG#11 | 0.788000 | 29 | 0.83883871 |
| *TLR2* CpG#4 | 0.924000 | 30 | 0.95287500 |
| *TLR2* CpG#10 | 1.000000 | 31 | 1.00000000 |
